# Preanalytical framework for routine clinical use of liquid biopsies: combining EVs and cfDNA

**DOI:** 10.20517/evcna.2025.44

**Published:** 2025-10-09

**Authors:** Nike K. Simon, Stefanie Volz, Jussara Rios de los Rios Reséndiz, Tatjana Wedig, Sophia H. Montigel, Nathalie Schwarz, Karsten Richter, Dominic Helm, Michelle Neßling, Lin Zielske, Julia Berker, Sophia Russeck, Monika Mauermann, Wolf-Karsten Hofmann, Stefan M. Pfister, Kristian W. Pajtler, Kendra K. Maaß, Katharina Clemm von Hohenberg

**Affiliations:** ^1^Division of Pediatric Neurooncology, German Cancer Consortium (DKTK), German Cancer Research Center (DKFZ) Heidelberg, Heidelberg 69120, Germany.; ^2^Hopp Children’s Cancer Center Heidelberg (KiTZ) and NCT Heidelberg, Heidelberg 69120, Germany.; ^3^Medical Faculty, Heidelberg University, Mannheim 68167, Germany.; ^4^Department of Pediatric Oncology, Hematology and Immunology, Heidelberg University Hospital, Heidelberg 69120, Germany.; ^5^Junior Clinical Cooperation Unit Translational Lymphoma Research, German Cancer Research Center (DKFZ) Heidelberg, Mannheim 68169, Germany.; ^6^Department of Hematology and Oncology, Medical Faculty Mannheim, Heidelberg University, Mannheim 68167, Germany.; ^7^Core Facility Electron Microscopy, German Cancer Research Center (DKFZ) Heidelberg, Heidelberg 69120, Germany.; ^8^Proteomics Core Facility, German Cancer Research Center (DKFZ) Heidelberg, Heidelberg 69120, Germany.

**Keywords:** EV preservation, liquid biopsy, extracellular vesicles, cfDNA, human plasma, translation, isolation protocol, preanalytics

## Abstract

**Aim:** Liquid biopsies hold significant potential for the minimally invasive diagnosis of tumors and other diseases. While the clinical application of cell-free DNA (cfDNA) methodologies is emerging, the implementation of tumor-derived extracellular vesicles (EVs) as validated biomarkers is hindered by substantial preanalytical variations. In this work, we standardized the preanalytical procedures of blood collection for subsequent serial isolation of plasma cfDNA and EVs from a single blood collection tube.

**Methods:** We compared the impact of blood preservation tubes and storage to enable proteomic profiling of resulting EVs in addition to cfDNA extraction and sequencing. Following a stringent method of large EV (lEV) and small EV (sEV) isolation, consisting of differential ultracentrifugation and size exclusion chromatography, we evaluated the protein concentration, particle number, quality and integrity of the isolated EVs. Subsequent proteomic analyses of EV isolates revealed the complexity of the respective tube-biased proteomes, allowing the interpretation of EV origins as well as contamination sources.

**Results:** While ACD-A and Citrate tubes yield satisfactory results in the preservation of EV proteomes, only Streck RNA, Norgen, and PAX tubes can maintain high cfDNA purity for up to 7 days. When aiming for multiomics analyses, Streck RNA tubes showed the most stable performance across the tested parameters for both bioanalytes. Furthermore, we detected greater variability in protein composition in sEVs than in lEVs after 7 days of storage; thus, sEVs might be more susceptible to storage effects.

**Conclusion:** Our clinically applicable workflow provides the basis for informed choice of liquid biopsy tubes along with a ready-to-use protocol to retrieve both genomic and EV proteomic biomarker information for multiomics biomarker-based liquid biopsy studies.

## INTRODUCTION

The use of liquid biopsy, as an alternative to conventional and interventional biopsy, has recently started to revolutionize the diagnostic fields of diseases such as cancer, diabetes, and conditions detected through prenatal screening^[1-3]^. While liquid biopsies can be performed on any body fluid, such as urine, blood, cerebrospinal fluid, or breast milk, they are most commonly analyzed from peripheral venous blood^[4]^.

The technical term liquid biopsy comprises the analysis of various biomarkers, such as circulating tumor cells (CTCs), cell-free DNA (cfDNA), and extracellular vesicles (EVs), as well as circulating (mi)RNA or proteins^[5]^. While the preanalytical parameters for the analysis of nucleic acids have been studied extensively^[6-8]^, the analysis of proteins from EVs is still in its infancy. In addition, the purification of EVs poses significant technical challenges^[9]^, which hinder their easy application in clinical practice.

EVs can be subdivided into different categories, the functions of which are, however, still subject to study^[10]^. While apoptotic bodies (100-5,000 nm) arise from dying and dissolving cells, large EVs (also termed microvesicles, appr. 150-1,000 nm, lEVs) and small EVs (also termed exosomes, appr. 30-120 nm, sEVs) are thought to be vital for the intercellular communication of living cells, particularly tumor cells^[11]^. In their cells of origin, EVs are loaded with cargo consisting of RNA, DNA, proteins, and metabolites. They are either secreted as sEVs through the release of multivesicular body (MVB) or formed as sEVs via direct budding from the plasma membrane (lEVs)^[10]^. EVs can be taken up by different target cells and therefore serve as communication hubs^[12]^. Under specific physiological or pathological conditions, such as systemic inflammation, pregnancy, or cancer, the quantity of plasma EVs is significantly increased^[13-15]^.

While the field of EV research has greatly expanded owing to its diagnostic and therapeutic potential, some practical questions of standardization must be addressed before routine clinical application. One key issue is the choice of anticoagulants for blood sampling and storage before downstream analysis.

After blood draws, blood cells undergo hemolysis or cell death in a time- and temperature-dependent manner^[16]^. The choice of anticoagulants or preserving reagents can help reduce these processes^[17,18]^. Previous studies focusing on plasma and serum proteomes have highlighted the significant impact of sampling and processing variables on biomarker discovery. Moreover, standardized preanalytical protocols have been developed to facilitate the combined analysis of proteomes and metabolomes from plasma and serum samples and provide reference data sets. However, these studies did not incorporate the isolation or analysis of EVs^[19,20]^. Physiologic EV populations in the peripheral blood are contaminated after sampling by EVs from other cellular components of full blood, mainly leukocytes, erythrocytes, and platelets, as the latter continue to shed extracellular vesicles *ex vivo*^[21]^. If not considered carefully, these processes could lead to a change in the EV population and, therefore, EV protein content, ultimately resulting in the dilution of physiological EVs, which in turn impedes the accuracy and interpretability of biomarker studies. Awareness of preanalytical biases is fundamental for the interpretation of liquid biopsy sample composition. One crucial preanalytical factor is the choice of blood collection tube and the impact of anticoagulants or preservation chemistry on EV composition during storage before processing and analysis. The most commonly used tubes for liquid biopsy in routine clinical diagnostics are EDTA- or Citrate-supplemented tubes^[22]^. However, these tubes necessitate immediate processing of the blood sample through centrifugation for plasma separation at the site of patient care, which is often not feasible in clinical practice owing to limited time, personnel, and equipment. Alternatively, preserving formulations stabilize peripheral blood cells to facilitate subsequent liquid biopsy analysis and can, according to the manufacturer’s instructions, be left at room temperature (RT) for several days before processing^[23]^. However, thus far, they have mostly been used for cfDNA and have not been thoroughly tested for other applications, such as EV analyses. More recently, specialized tubes for EV research have entered the market and claim their compatibility with EV research.

To this end, our study aimed to compare five different preservation tubes (ACD-A, Norgen, PAX, Streck RNA, Streck DNA) and EDTA tubes as a reference 0-day baseline to minimize potential confounding factors. Furthermore, we included citrate-supplemented tubes (Citrate) in our study, as they are frequently used in EV studies, are readily available in clinical practice, and are comparably cost-efficient. We assessed EV quantity, quality and EV-associated protein content as well as cfDNA quantity and quality from blood plasma after sampling and storage in different preservation tubes. We detected significant differences in EV and cfDNA quality among the tested preservation tubes, even when they were processed immediately. Although incubation at RT for several days generally reduced the quality of EV-derived proteomes, this effect was markedly attenuated in certain preservation tubes.

Among these, Streck RNA tubes exhibited the most consistent performance across all tested parameters for multiomics applications. Notably, protein composition variability was greater in sEVs than in lEVs after 7 days of storage, indicating that sEVs are more vulnerable to storage-induced changes. To facilitate rapid clinical adoption, we provide a streamlined workflow that supports the selection of optimal liquid biopsy tubes and includes a practical protocol for extracting genomic and EV proteomic biomarkers, paving the way for comprehensive multiomics liquid biopsy-based clinical studies.

In summary, this study lays the basis for an informed choice of liquid biopsy tube and interpretation of EV-derived data for clinical studies in EV and cfDNA research.

## METHODS

### Sample population

**Figure 1:**
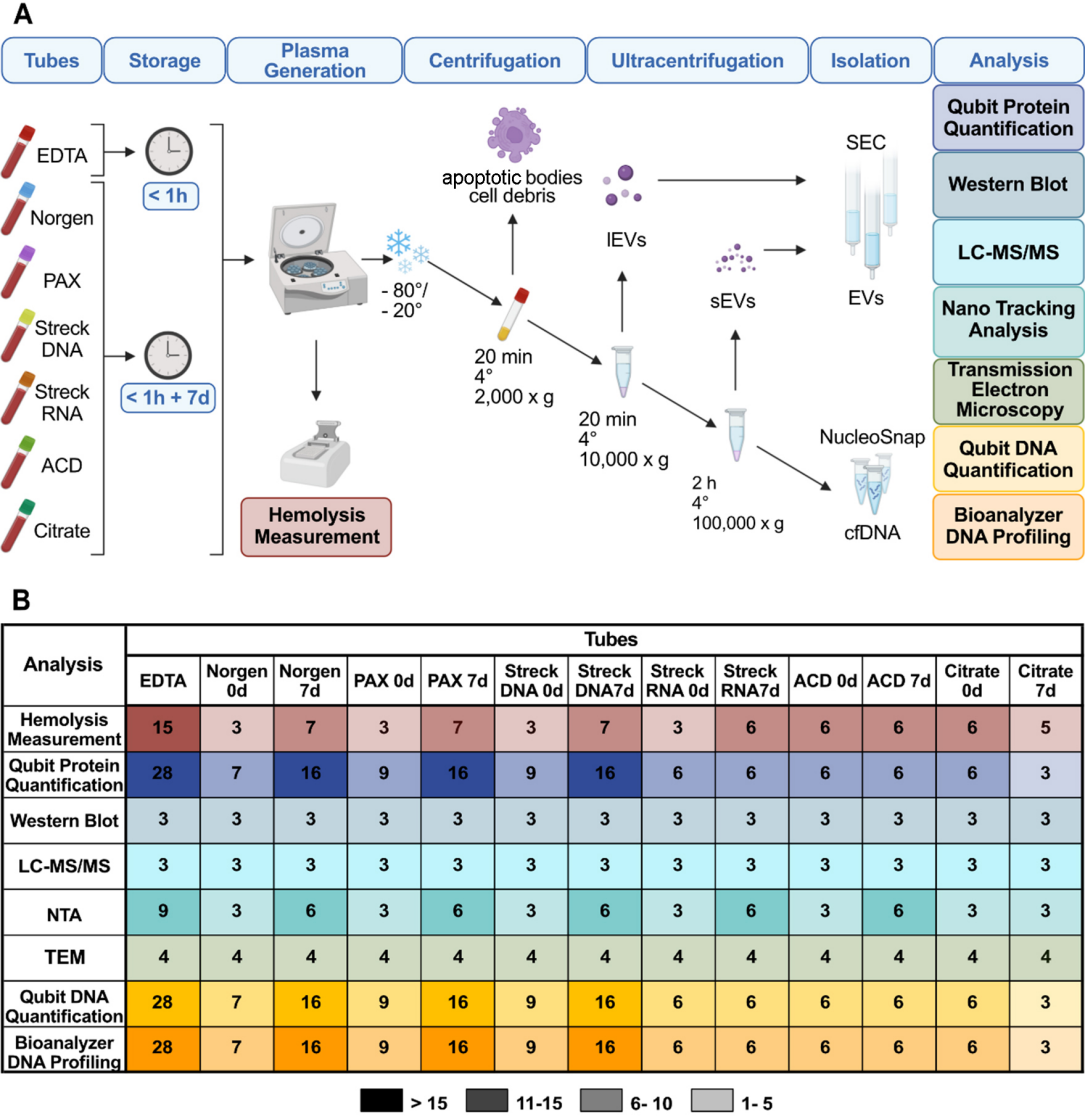
Overview of the tube comparison study design. (A) Outline of the experimental workflow comprising isolation of large EVs (lEVs) and small EVs (sEVs) via centrifugation, ultracentrifugation, size exclusion chromatography (SEC) and isolation of cell-free DNA (cfDNA). Evaluation of EDTA, Norgen, PAX, Streck DNA, Streck RNA, ACD, and Citrate tube performance after immediate processing (< 1 h) and long-term storage (7 days) via protein quantification, Western blot, nanoparticle tracking analysis (NTA), liquid chromatography-tandem mass spectrometry (LC-MS/MS), transmission electron microscopy (TEM), DNA quantification, and DNA fragment length analyses; (B) Summary of the study cohort illustrating the number of plasma samples collected from healthy individuals immediately after processing and after long-term storage in EDTA, Norgen, PAX, Streck DNA, Streck RNA, ACD, and Citrate for comparison of tube performances with hemolysis measurements (red), EV characterization (blue and green), and cfDNA analysis (yellow), including concentration and cfDNA purity. The numbers indicate replicates per condition. EVs: Extracellular vesicles.

**Figure 2:**
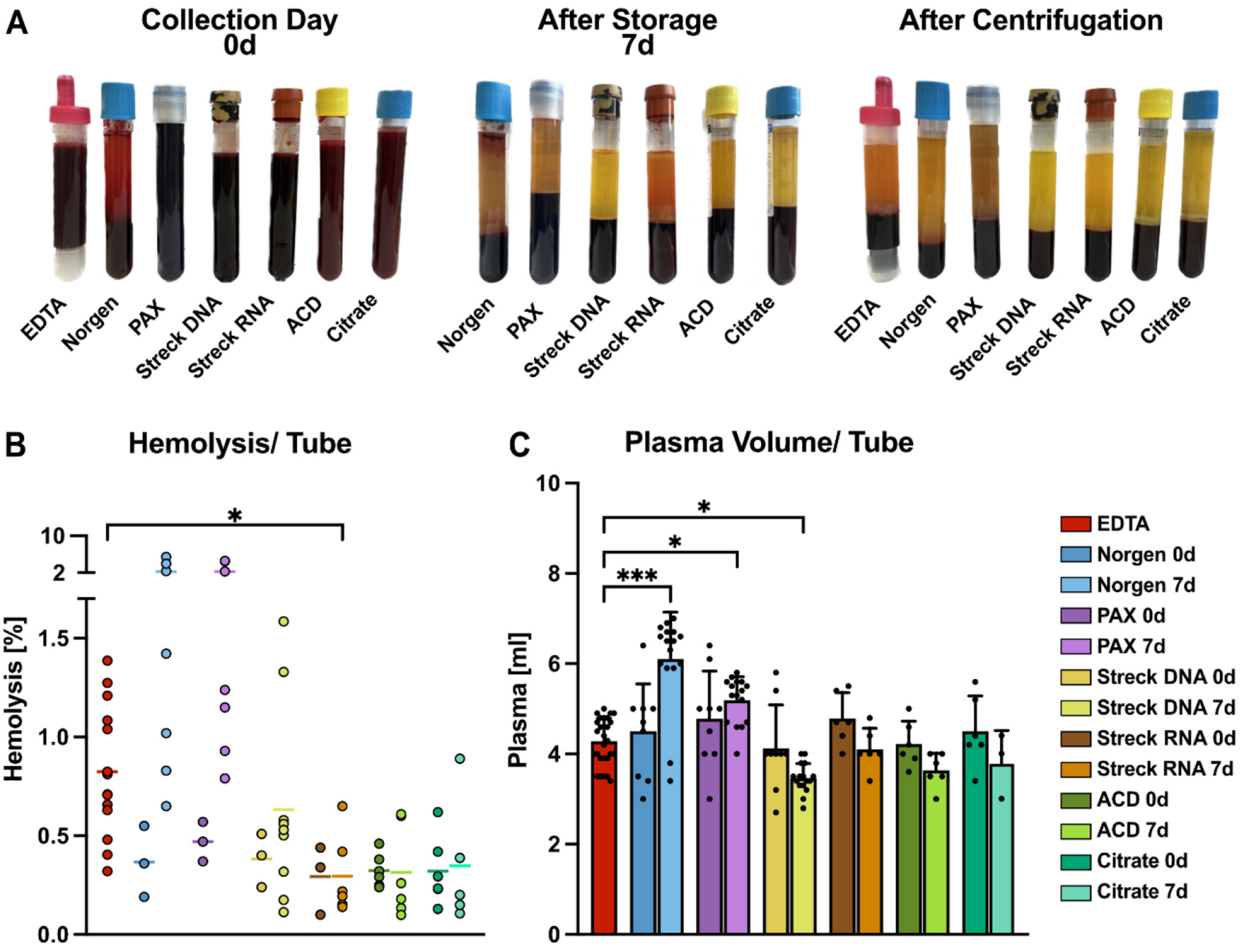
Comparative analysis of blood plasma collected from tube candidates. (A) Representative images of blood collection tubes, namely EDTA, Norgen, PAX, Streck DNA, Streck RNA, ACD-A, and Citrate, from one healthy donor for a direct comparison of tube appearance. Pictures were taken immediately after blood drawing (0 d, left), after 7 days of storage at RT (middle), and after plasma processing by centrifugation on day 0 (right); (B) Hemolysis levels determined by the relative levels of free hemoglobin measured as blood plasma absorbance at 414 nm; (C) Mean plasma volume obtained from the indicated blood tubes recovered after centrifugation. Statistical significance for (B) and (C) was determined for all tube candidates via the Kruskal-Wallis test, followed by Dunn’s multiple comparisons test. ^*^P < 0.05, ^***^P < 0.001. Only statistically significant differences compared with EDTA are shown in the graphs; nonsignificant results are not labeled. RT: Room temperature.

**Figure 3:**
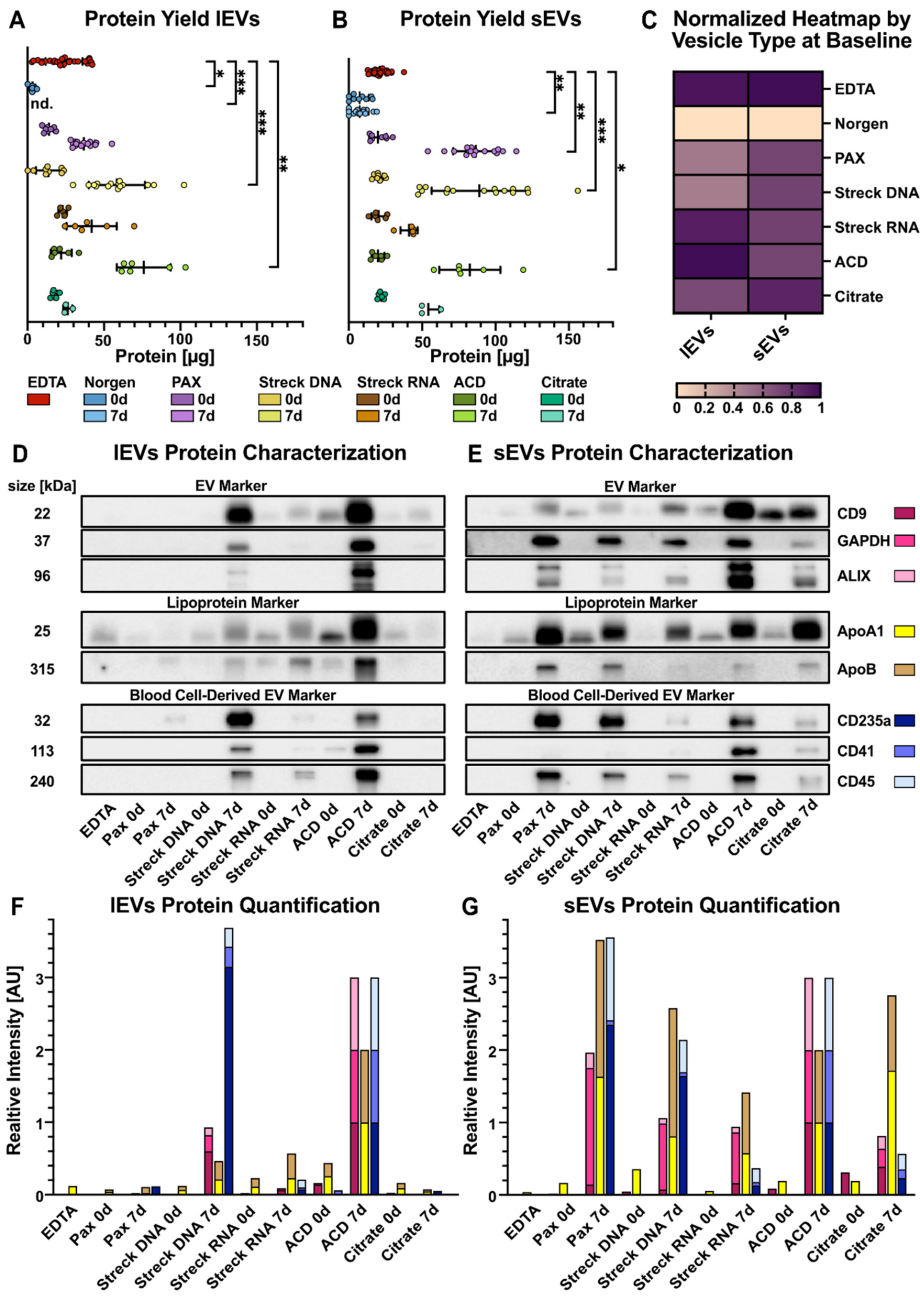
Quantitative analysis of lEVs and sEVs. (A) lEV and (B) sEV amounts quantified as the total amount of protein in the three peak SEC fractions combined, allowing for direct comparison; (C) Protein amounts of lEV and sEV peak fractions, normalized and internally scaled per vesicle type, in the indicated tubes after immediate processing; (D and E) Western blot analyses of pooled lEV and sEV peak fractions. EVs isolated from three healthy individuals were pooled in equal volumes prior to Western blotting to ensure representability. Representative blots of classical EV markers, lipoprotein contaminants, and blood-derived EV markers, which serve as qualitative indicators of EV identity and purity, are shown; (F and G) Western blot quantification of protein bands. Signal intensities were normalized to the corresponding ACD 7 d band to account for variations in protein loading and transfer. Norgen was excluded from the Western blot analyses [Figure 3D-G] because of the limited amount of protein. Statistical significance in (A) and (B) was determined for all tube candidates via the Kruskal-Wallis test, followed by Dunn’s multiple comparisons test. ^*^P < 0.05, ^**^P < 0.01, ^***^P < 0.001. Statistically significant differences compared with EDTA are shown in the graphs; nonsignificant results are not labeled. EVs: Extracellular vesicles; lEVs: large EVs; sEVs: small EVs.

**Figure 4:**
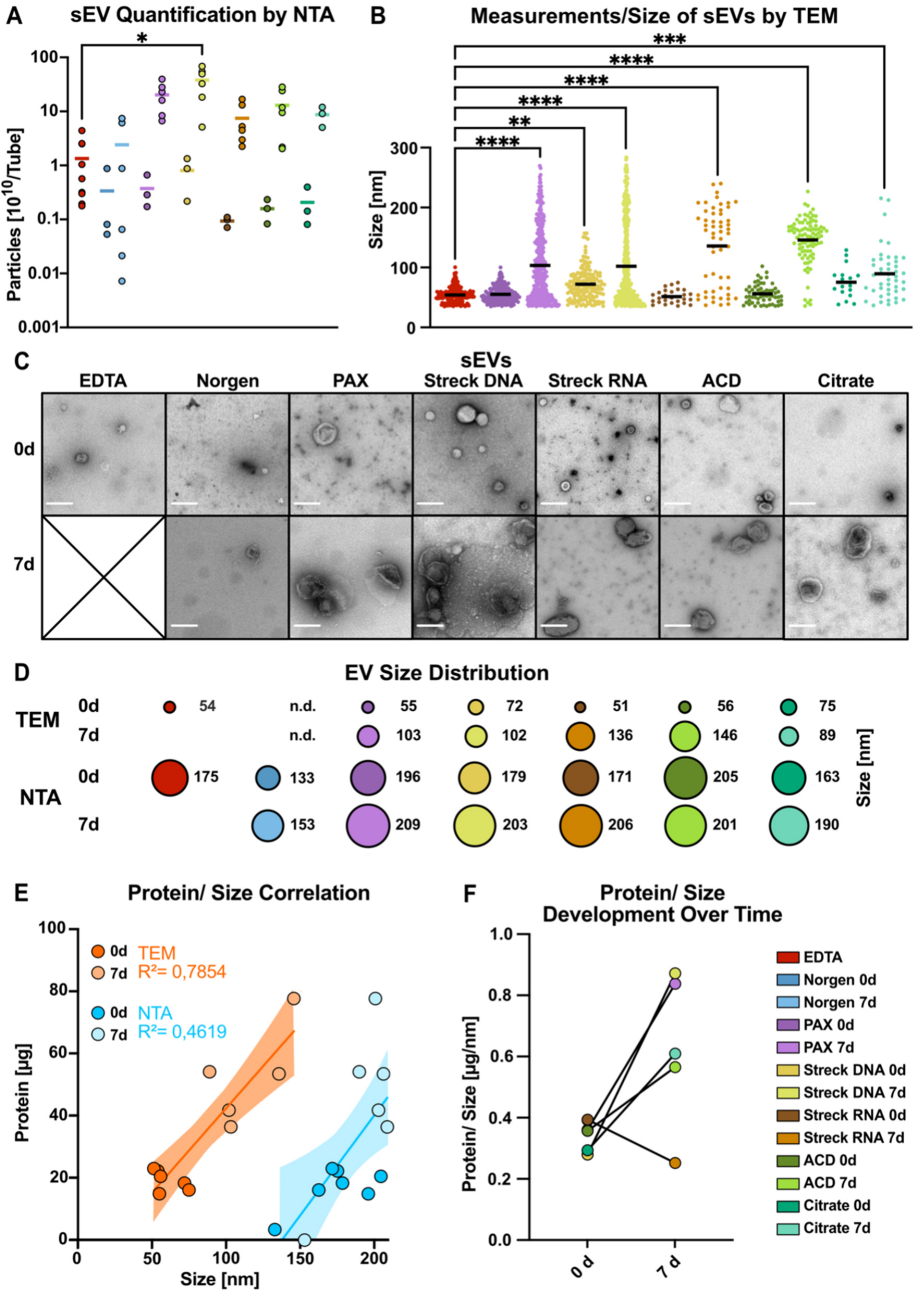
Methodological comparison of the size and integrity of sEVs. (A) NTA quantification of particle numbers in three pooled sEV peak fractions; (B) sEV particle diameter measured via negative EM staining; (C) Negative-staining electron microscopy (TEM) image of sEVs after immediate processing (< 1 h) and after long-term storage for 7 days (scale bar = 200 nm); (D) The mean size of sEVs was measured via negative EM staining (top) and NTA (bottom); (E) Correlations of protein contents and estimated sEV sizes measured via EM images (dark orange, 0 d; light orange, 7 d) and NTA images (dark blue, 0 d; light blue, 7 d) for all the tube candidates; (F) Changes in the protein-to-size ratio of sEVs plotted for immediately processed samples and after 7 days of storage in the indicated tubes. Norgen did not meet the threshold and was therefore excluded from the EM analysis [Figure 3B and F]. The color code in (F) applies to all the graphs in this figure. Statistical significance for (A) was determined for all tube candidates via the Kruskal-Wallis test, followed by Dunn’s multiple comparisons test. ^*^P < 0.05; (B) ANOVA was used for multiple comparisons, followed by the Sidak test. ^**^P < 0.01, ^***^P < 0.001, ^****^P < 0.0001. Only statistically significant differences compared with EDTA are shown in the graphs; nonsignificant results are not marked; (E) The linear regression line is shown with 95% confidence intervals, *R*^2^, as depicted, *P* = 0.0106 for NTA and *P* = 0.0003 for EM. EVs: Extracellular vesicles; sEVs: small EVs; NTA: nanoparticle tracking analysis.

To evaluate the performance of the six tube candidates compared with 0-day baseline EDTA tubes, we collected blood from 10 healthy individuals, 8 women and 2 men aged between 23 and 48 years. Mass spectrometric and Western blot analyses were performed on time-matched samples from three healthy volunteers (two females and one male). Healthy volunteers were of Caucasian ethnicity due to availability. Each sample had a matched EDTA sample that was processed immediately and served as the 0-day baseline for normalization [Figures 1 and 2]. For analyses of protein amount via Qubit protein measurements [Figure 3] as well as particle amount and size measurements via NTA and EM images [Figure 4], data from the entire sample cohort were included [Figure 1B].

**Figure 5:**
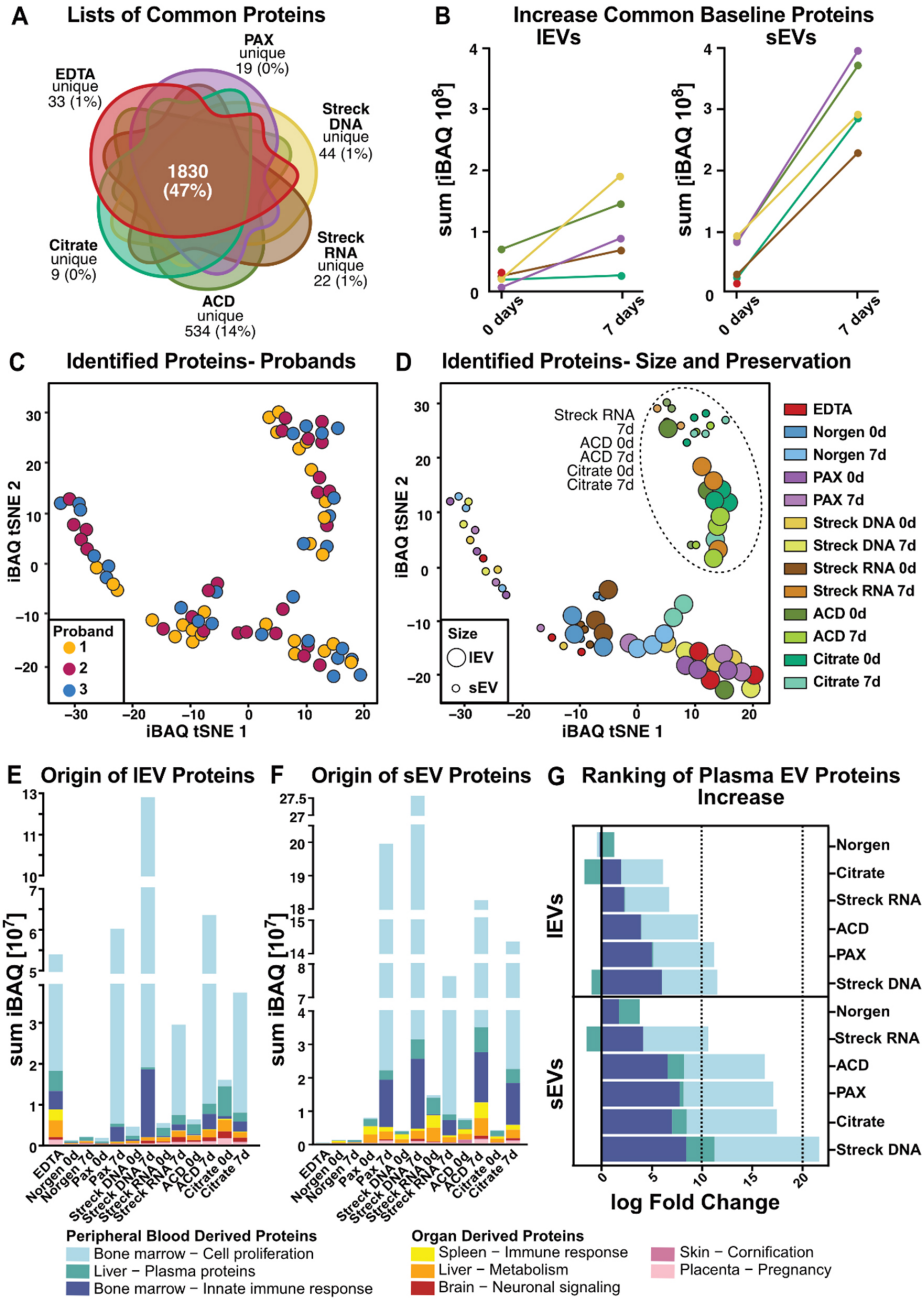
Comprehensive characterization of the lEV and sEV proteomes. (A) Venn diagram illustrating the overlap of mass spectrometry-identified proteins per tube. Norgen was excluded because it did not meet the threshold due to insufficient sample quantity; (B) Increase in standard 0-day baseline EV protein iBAQ values over time; (C) t-SNE clustering with participant individual color codes. Each dot represents the iBAQ proteome values of individual EV samples, with each participant indicated by different colors or (D) with processing time (0 d or 7 d) and blood tube (EDTA, Norgen, PAX, Streck DNA, Streck RNA, ACD, and Citrate) indicated. (E and F) Tissue origin predictions for (E) lEV and (F) sEV iBAQ protein values; (G) iBAQ log fold change of plasma proteins (defined as bone marrow-cell proliferation, liver-plasma proteins and the bone marrow-inorganic immune response) during storage for 7 *vs.* 0 d. Norgen was excluded from Figure 5A and B because it fell below the threshold due to the limited protein amount isolated. EV: Extracellular vesicle; lEVs: large EV; sEVs: small EV; t-SNE: t-distributed stochastic neighbor embedding; iBAQ: intensity-based absolute quantification.

For further proteomic analyses via Western blot [Figure 3] and mass spectrometry [Figure 5], samples from both time points (0 and 7 days) were collected simultaneously to ensure physiological comparability and to minimize confounding factors, such as stress or metabolic effects.

Informed consent was obtained from all participants, with protocols approved by Heidelberg University (ethics approval ID: *S795/2020*), and the procedures were performed in accordance with the Declaration of Helsinki.

### Plasma isolation

Peripheral whole blood was collected via standard venipuncture via a 21 G safety-multifly needle (Sarstedt, Nümbrecht, Germany). Blood was drawn directly into three anticoagulation tubes without cell-stabilizing additives, namely 9 mL K3EDTA S-monovettes® (Sarstedt, Nümbrecht, Germany), 3 × 3 mL S-Monovette® Citrate 3.2% (Sarstedt, Nümbrecht, Germany) and Vacuette® tube 9 mL Trinatriumcitrat 3.2% (Greiner Bio One, Frickenhausen, Germany), and into cell-stabilizing tubes, namely 8.7 mL cf-DNA/cf-RNA Preservative Tubes from Norgen (Norgen Biotek, Thorold, ON, Canada), 10 mL PAXgene Blood ccfDNA Tube (CE-IVD, PreAnalytiX, Qiagen, Hilden, Germany), 10 mL cell-free DNA BCT tubes (Streck, La Vista, NE, USA), 10 mL RNA Complete BCT® tubes (Streck, La Vista, NE, USA), and Vacuette® Röhrchen 9 mL ACD-A (Greiner Bio One, Frickenhausen, Germany) [Supplementary Table 1].

All the tubes were filled until the vacuum was exhausted. The tubes were inverted immediately after blood collection according to the manufacturer’s recommendations. The EDTA tubes were centrifuged within 1 h of blood collection. Blood drawn into the tested tube candidates was centrifuged either within 1 h or left at RT for 7 days. The blood was centrifuged according to the manufacturer’s instructions to produce platelet-poor plasma (PPP). After centrifugation, the plasma was collected to at least 0.5 cm above the buffy coat. The PPP volume was measured, and the samples were aliquoted into 15 mL TPP^TM^ tubes (Merck SA, Germany) and stored at -20 °C until further use.

### Differential centrifugation

Before further processing, the plasma was thawed slowly on ice and centrifuged at 2,000 × *g* for 20 min at 4 °C in a Heareus^TM^ Varifuge 3.0 R (Heraeus, Hanau, Germany) to remove larger apoptotic bodies and cellular debris. Prior to use, ultracentrifugation tubes (Beranek, USA, 326819) were incubated with 70% ethanol for 10 min, which was removed before further use. 2,000 × *g* supernatants were directly transferred into ethanol-pretreated and air-dried ultracentrifugation tubes and centrifuged at 10,000 × *g* for 20 min at 4 °C in a Beckman Optima L8-70 M ultracentrifuge (Beckman Coulter GmbH, Krefeld, Germany) using a 55 Ti swing-bucket rotor with breaks activated. The resulting 10,000 × *g* (10 K) pellets were resuspended in 100 µL of 0.22 µm-filtered PBS to obtain the lEV fraction. The supernatants were transferred into freshly ethanol-treated ultracentrifugation tubes, followed by ultracentrifugation at 100,000 × *g* for 2 h. The resulting 100,000 × *g* (100 k) pellets were resuspended in 100 µL of 0.22 µm-filtered PBS to obtain the sEV fraction. The supernatant from the 100 K ultracentrifugation was subjected to cfDNA isolation and stored at -20 °C until further use. Both 10 K pellets containing the lEV fraction and 100 K pellets containing the sEV fraction were stored at -20 °C in protein low-binding tubes (Sarstedt, Nümbrecht, Germany) [Figure 1].

### Hemolysis measurements

Hemolysis was quantified from plasma aliquots via a NanoDrop One Microvolume UV-Vis Spectrophotometer (Thermo Fischer Scientific, Waltham, MA, USA) by measuring the absorbance at 414 nm. A standard curve was generated starting from a fully lysed red blood cell sample, and serial dilutions ranging from 100% to 0.025% were prepared. Standard curve and sample measurements were performed using a 2 µL volume. The degree of hemolysis was calculated within the linear range of the standard curve [Figure 2, Supplementary Figure 1].

### Size exclusion chromatography

Single qEV Legacy 35 nm columns (Izon, Christchurch, New Zealand) were allowed to reach RT for 30 min. First, the columns were flushed with at least one column volume of 0.22 µm-filtered PBS. Resuspended 10 and 100 K pellets (100 µL each) were added to individual columns. As soon as the sample volume had entered the column, 0.22 µm-filtered PBS was added to the top of the column, and collection of the first void volume fraction (F0, 900 µL = void volume of the column) was started. The F1 to F9 fractions (100 µL each) were collected in 1.5 mL Eppendorf tubes (Eppendorf, Hamburg, Germany) according to the manufacturer’s instructions.

### Protein quantification

The protein concentration of each SEC fraction (F0-F9) was measured via a Qubit protein assay kit (Thermo Fisher Scientific, Waltham, MA, USA). Briefly, a working solution containing the provided Qubit^TM^ protein buffer and Qubit protein reagent was prepared, and a standard curve was generated following the manufacturer’s protocol. All the samples were vortexed with 0.1% SDS for at least 1 min to ensure lysis of the EVs and release of luminal EV proteins. The sample measurement was performed using a sample input of 8 and 192 µL of Qubit working solution. For each round, the buffer threshold of the negative controls was defined. After an incubation time of 15 min at RT, all the fractions were measured on a Qubit 3.0 fluorometer (Thermo Fisher Scientific, Waltham, MA, USA) with a detection range between 12.5 μg/mL and 5 mg/mL. The protein peak fractions were combined or analyzed separately (as indicated in the respective figures), and the total protein yield was calculated.

For the protein quantification of apoptotic bodies (2,000 × *g* pellet), we used BCA measurements instead of Qubit measurements because of the high protein concentration in these samples. The samples were lysed in 0.1% SDS (w/v) and subjected to protein quantification via the Pierce^TM^ BCA Protein Assay Kit (Thermo Fisher Scientific) according to the manufacturer’s instructions. Depending on the expected protein yield, samples were diluted either 1:10 or 1:100 in PBS prior to measurement. The absorbance was recorded at 562 nm via a plate reader (e.g., SpectraMax or equivalent), and protein concentrations were calculated on the basis of a BSA standard curve. Each time point and tube was measured in three biological duplicates.

### Western blotting

Three replicates of sEV samples from each of the two different time points were stored in Laemmli buffer for immunoblotting analysis. Owing to the limited protein yield from Norgen tube samples, these samples were excluded from Western blot analysis. To obtain comparable and representative data, as shown in Figure 3, EV protein lysates from three volunteers were pooled into a single composite sample per time point. In Supplementary Figure 2, peak fractions were pooled and subjected to ultrafiltration via Amicon Ultra15 Centrifugal filter 10 kDa (Merck Millipore, Darmstadt, Germany) and centrifugation at 3,683 × *g* at 4 °C for 20 min. Equal sample volumes were then loaded on custom-made SDS-PAGE gels and transferred to a 0.45 µm PVDF membrane (Immobilion-P, Merck Millipore, Germany). After Ponceau staining, the membranes were incubated in 5% skim milk PBST (PBS containing 1% Tween-20) for 20-60 min, briefly rinsed with PBST, and then incubated with primary antibody solution (5% skim milk PBST) overnight at 4 °C. The membranes were then washed three times, 15 min each, in PBST before incubation in secondary antibody solution [1:10,000 in 5% skim milk (PBST)] for one hour at RT. Afterwards, the secondary antibodies were removed by washing three times for 5-15 min each. Finally, chemiluminescence was detected via the use of Supersignal West Pico & Femto ECL reagents (Thermo Fischer Scientific, Life Technologies, USA) and a Bio-Rad ChemiDoc Imaging System (Bio-Rad Laboratories, Feldkirchen, Germany) [Figure 3, Supplementary Figure 2].

### Antibodies

For Western blotting, the following primary antibodies were used: CD9 (Cell Signaling Technology, 13403, 1:1,000), GAPDH (Proteintech, 60004-1, 1:1,000), ALIX (Abcam, ab275377, 1:1,000), ApoA1 (R&D Systems, MAB36641, 1:2,500), ApoB (R&D Systems, MAB41242, 1:1,000), CD235a (Invitrogen, JC159, 1:500), CD41 (Abcam, ab83961, 1:1,000), and CD45 (Abcam, ab281586, 1:1,000). The following secondary antibodies were used: peroxidase-conjugated AffiniPure goat anti-mouse IgG (Jackson ImmunoResearch Laboratories, 115-035-003, 1:10,000) and peroxidase-conjugated AffiniPure goat anti-rabbit IgG (Jackson ImmunoResearch Laboratories, 111-035-003, 1:10,000).

### Particle quantification

Particle quantification of sEV samples was performed via NTA via a NanoSight LM10 instrument equipped with a 405 nm laser (Malvern Instruments, Malvern, UK). The samples were adjusted to protein concentrations and diluted 1:500 to 1:1,000 in 0.22 µm-filtered PBS for NTA. The camera level and detection threshold were set to 13 and 5, respectively. The background was assessed by measuring 0.22 µm-filtered PBS. For each sample, three videos of 30 s each were recorded and analyzed via NTA version 3.0 software (Malvern Instruments, Malvern, UK) [Figure 4].

### Transmission electron microscopy

The sEV-containing SEC peak fraction, identified via the Qubit protein measurements, was adsorbed onto glow-discharged carbon-coated grids (Electron Microscopy Science), washed in aqua bidest, and negatively stained with 2% aqueous uranyl acetate (Merck, Darmstadt). Micrographs were taken with a Zeiss EM 910 at 80 kV (Carl Zeiss, Oberkochen, Germany) via a slow-scan CCD camera (TRS, Moorenweis, Germany).

For transmission electron microscopy (TEM) image analyses, we first applied segmentation of representative TEM images via pixel classification via ilastik version 1.4.0 software^[24]^. Subsequently, probability maps were generated from five technical replicates each, which were measured (cutoff at 35 nm diameter) and further analyzed via ImageJ [Figure 4, Supplementary Figure 3].

### Liquid chromatography-tandem mass spectrometry

Isolated EVs dissolved in PBS were lysed via the above-described lysis protocol.

Proteins (2 µg) were digested (trypsin) via an AssayMAP Bravo liquid handling system (Agilent Technologies) running the autoSP3 protocol according to Müller *et al.*^[25]^.

Liquid chromatography-tandem mass spectrometry (LC-MS/MS) analysis was carried out on a Vanquish Neo system (Thermo Fisher Scientific) directly connected to an Orbitrap Exploris 480 mass spectrometer for a total of 60 min. Peptides were desalted online on a trapping cartridge (Acclaim PepMap300 C18, 5 µm, 300 Å wide pore; Thermo Fisher Scientific) for 3 min with a 30 µL/min flow of 0.05% TFA in water. An analytical multistep gradient (300 nL/min) was applied via a nanoEase MZ Peptide analytical column (300 Å, 1.7 µm, 75 µm × 200 mm; Waters) with solvent A (0.1% formic acid in water) and solvent B (0.1% formic acid in acetonitrile). For 46 min, the concentration of B was linearly increased from 2% to 30%, followed by a quick increase to 80%. After 4 min, the concentration of B was decreased to 2%, and an equilibration step (three column volumes) was applied. Eluting peptides were analyzed via mass spectrometry in data-independent acquisition (DIA) mode^[26]^. A full scan at 120 k resolution (380-1,400 m/z, 300% AGC target, 45 ms maxIT) was followed by 20 windows of variable masses for fragment spectra acquisition covering the mass range of 400-1,000 m/z with 1 Da overlap (30 k resolution, AGC target 1,000%, maxIT 54 ms, collision energy 28%). To minimize the risk of cross-sample contamination, each sample was subjected to a wash run (40 min). Instrument performance throughout the course of the measurement was monitored by regular (approximately one per 48 h) injections of a standard sample and an in-house bioinformatic application.

Analysis of DIA RAW files was performed with Spectronaut (Biognosys, version 17.1.221229.55965) in directDIA+ (deep) library-free mode^[27]^. Default settings were applied with the following adaptations. Within the Pulsar Search in Peptides, the Max Peptide Length was set to 35. In the results filters, the peptide charge was enabled, the maximum charge was set to 6, and the minimum charge was set to 2. For DIA analysis under identification, the precursor PEP cutoff was set to 0.01, the protein Q value cutoff (Run) was set to 0.01, and the protein PEP cutoff was set to 0.05. For quantification, the proteomics filter was set to only protein group-specific, cross-run normalization was disabled, the quantification window was set to “not synchronized”, and the major group quantity was defined as the sum of peptide quantities. The data were searched against the human proteome from UniProt^[28]^ (a human reference database with one protein sequence per gene, containing 20,591 unique entries from March 1, 2023) and the contaminant FASTA from MaxQuant^[29]^ (246 unique entries from December 22, 2022). Fractions were set to enable separate normalization for small and large EVs.

All bioinformatic analyses were performed via R version 4.1.3^[30]^. To ensure the reliability and relevance of the results, contaminants, including trypsin (used for protein digestion in mass spectrometry), keratins, and proteins identified as *Bos taurus*, were excluded from subsequent analyses. Protein quantification was carried out via intensity-based absolute quantification (iBAQ)^[31]^. The iBAQ method quantifies protein abundance by summing the intensities of all identified peptides for each protein and then normalizing them based on the number of theoretically observable tryptic peptides [Figure 5, Supplementary Figures 4 and 5, Supplementary Tables 2-11]. For downstream analysis, either the full list of identified proteins (excluding contaminants, Supplementary Table 9, Figure 5A, E and F, Supplementary Figure 4A, B and E-M, Supplementary Figure 5A) or the following specific subsets were utilized: the common 0-day baseline protein list [Figure 5B-D, Supplementary Table 2], the top EV protein list [Supplementary Figure 4C and D], the novel EV protein list [Supplementary Figure 4N], and the plasma-derived EV protein list [Supplementary Figure 5B and C, Supplementary Table 5]. An overview of all the Supplementary Tables and the corresponding figures is provided in Supplementary Table 11.

The common 0-day baseline protein list represents the overlap of all mass spectrometry-identified proteins across the different tubes on day 0, i.e., proteins that were detected in at least one of the three replicates per tube on day 0, excluding the Norgen tube, which was omitted owing to insufficient protein quantity. A total of 1,830 proteins were included in this list.

The top EV protein list consisted of 51 proteins from our full list of proteins that were listed in the top 100 EV-associated proteins according to the Vesiclepedia^[32]^ database [Supplementary Table 4], whereas the novel EV protein list contained all proteins in our full analyses that had not already been described in either the Exocarta^[33]^ or the GO^[34,35]^ database.

Finally, the plasma-derived EV protein list was created by cross-referencing the proteins identified in our study with those reported in the literature, specifically by Vallejo *et al.*^[36]^ and Dhondt *et al.*^[37]^, resulting in a total of 1,346 proteins. The specific protein list used for each analysis can be found in the corresponding Supplementary Table 5. To visualize the composition of the lists, a Venn diagram was constructed via the eulerr R package^[38]^. Additionally, dimensionality reduction was performed via t-SNE, implemented through the Rtsne package^[39-41]^, to visualize the clustering of the samples. Heatmaps were generated via the ComplexHeatmap package^[42,43]^ to visualize the top 100 EV proteins, as reported in the Vesiclepedia^[32]^ database.

To assess the change in the abundance of common 0-day baseline proteins over the course of the study (from day 0 to day 7) and to analyze the origin of the proteins, iBAQ values for each gene were averaged across the replicates per tube to obtain a mean value per tube. The tissue origin of EV proteins was determined by referencing the Human Protein Atlas^[44,45]^.

Data visualization throughout the analysis was carried out via the ggplot2^[46]^ and ggbreak R packages^[47]^.

### cfDNA isolation

cfDNA was coisolated from 100 K supernatants of the corresponding EV isolation process. The supernatants were thawed on ice, and cfDNA was isolated via the NucleoSnap cfDNA Kit (Macherey-Nagel, Düren, Germany) following the manufacturer’s protocol. The final elution step was performed in 50 µL of nuclease-free water (nf-H_2_O; Thermo Fisher Scientific, Waltham, MA, USA) after 5 min of membrane incubation. 6 µL cfDNA eluate was aliquoted for quality control measurements. The samples were stored at -20 °C until further analysis.

### cfDNA quantification and quality control

The DNA concentration was assessed via a Qubit dsDNA HS Assay Kit (Thermo Fisher Scientific, Waltham, MA, USA) on a Qubit 3.0 fluorometer (Thermo Fisher Scientific, Waltham, MA, USA). The detection range of the Qubit dsDNA HS Assay Kit (Thermo Fisher Scientific, Waltham, MA, USA) is 0.1-120 ng/mL.

To assess the amount of cfDNA as well as gDNA contamination, the Bioanalyzer High-Sensitivity DNA Kit (Agilent, Santa Clara, CA, USA) was used according to the manufacturer’s instructions, with 1 µL per sample. Samples with a concentration of > 1.0 ng/µL were diluted to a final concentration of 1.0 ng/µL to match the optimal performance range of the Bioanalyzer Chips. The results were analyzed via the 2100 Bioanalyzer Expert software (Agilent Technologies, Santa Clara, CA, USA). Fragments between 150 and 500 bp in length were considered cfDNA, and the remaining fragments within the range of 50-7,000 bp were considered contaminants with gDNA.

### Statistical analysis

Data management and calculations were performed via GraphPad Prism 9 (GraphPad Software, Inc., La Jolla, CA, USA). Comparisons between two groups were performed via the Wilcoxon matched-pairs signed-rank test and the unpaired *t*-test. For comparisons of more than two groups, the Kruskal-Wallis test, followed by Dunn’s multiple comparisons test or ANOVA followed by the Sidak test, was performed. The statistical significance of the performance of blood preservation tubes was always tested against EDTA values. p values of < 0.05 were considered statistically significant, with *P* values represented as follows: ^*^P < 0.05, ^**^P < 0.01, ^***^P < 0.001, ^****^P < 0.0001. “ns” denotes differences in means that were not significant. All error bars shown represent standard deviations unless otherwise stated.

### EV TRACK

We have submitted all the relevant data from our experiments to the EV-TRACK knowledgebase (EV-TRACK ID: EV250048) (Van Deun J, *et al.* EV-TRACK: transparent reporting and centralizing knowledge in extracellular vesicle research. Nature methods. 2017;14(3):228-32)^[48]^.

You may access and check the submission of experimental parameters to the EV-TRACK knowledgebase via the following URL: http://evtrack.org/review.php. Please use the EV-TRACK ID (EV250048) and the last name of the first author (Simon, Volz) to access our submission.

## RESULTS

### Study design

To advance multiomics liquid biopsy, specifically combinatorial analyses of EVs and cfDNA, toward clinical application, we aimed to compare different blood preservation tubes. We tested two conventional (EDTA, Citrate) and five specialized liquid biopsy tubes (Norgen, PAX, Streck DNA, Streck RNA, ACD-A) [Figure 1A, Supplementary Table 1]. EDTA tubes were always processed within one hour from the blood draw as a reference for normalization, whereas for all other tubes, one was processed immediately, and one was left at RT for seven days before processing.

To recover PPP, we performed centrifugation following the manufacturer’s instructions for each tube. To simulate a real-world clinical-translational scenario in which EV isolation cannot be performed within the same day as sample collection, the PPP was stored at -20 or -80 °C. EV isolation was subsequently performed as described in the methods section, including thorough quality control assessment of both EVs and cfDNA, as graphically illustrated [Figure 1A]. All the purifications were performed from three to 28 samples per condition [Figure 1B], as indicated in the Methods Sample Population.

### The preservation tube and storage time affect hemolysis and the plasma volume

*Ex vivo* hemolysis in blood collection tubes has been described previously to affect the distribution and content of isolated EVs^[37]^. Therefore, we assessed the effects of the respective tube chemistries on hemolysis levels. Following visual inspection [Figure 2A], the plasma absorbance at 414 nm was used to quantify hemolysis. On day 0, the degree of baseline hemolysis on the collection day was greater in the EDTA tubes than in all the other preservation tubes [Figure 2A and B]. After storage for seven days at RT, we detected a tendency toward increased hemolysis levels in Norgen and PAX tubes [Figure 2A and B]. In contrast, the Streck RNA, ACD-A, and Citrate tubes presented significantly lower hemolysis levels than did the EDTA tubes [Figure 2B]. Significantly increased plasma volumes were observed in Norgen and PAX tubes, whereas significantly lower plasma volumes were recovered from Streck DNA after 7 days [Figure 2C]. Interestingly, the observed differences in plasma volume did not correlate with hemolysis levels [Supplementary Figure 1A], indicating that the changes in plasma recovery are unlikely to be solely explained by hemolysis-induced fluid shifts.

### Preservation tubes and storage time affect EV protein yields and contamination

Protein measurements from SEC fractions can aid in the discrimination of EV-enriched and soluble protein samples^[49]^. Therefore, we quantified the protein amounts of the EV peak fractions recovered from the tested tubes [Figure 3A-C, Supplementary Figure 2A]. To ensure that soluble, non-EV proteins were excluded from the analysis, only the EV-enriched peak fractions were selected based on their known elution profile. Prior to quantification, EVs were lysed with 0.1% SDS to release intravesicular proteins, allowing total EV-associated protein content to be measured. For quantification and further downstream analyses, the three peak fractions of sEVs and lEVs were combined [Figure 3A-C]. Exemplary SEC fraction protein profiles from PAX tubes on day 0 and after 7 days of preservation illustrate the selection process [Supplementary Figure 2B]. On day 0, we observed significantly lower protein yields in sEVs and lEVs isolated from Norgen tubes [Figure 3A-C, Supplementary Figure 2A]. Additionally, lEV fractions isolated from PAX tubes on day 0 yielded significantly less protein than did those isolated from EDTA tubes [Supplementary Figure 2A]. With the exception of Norgen tubes, on day 0, all other tubes presented EV quantities ranging from 14 to 24 µg per tube for both lEVs and sEVs. However, after 7 days, the overall protein yields of lEVs and sEVs increased in all the tested tubes. While this effect was observed for all the tubes, the smallest differences were detected in the Streck RNA and Citrate tubes. These results were consistent between lEVs [Figure 3A] and sEVs [Figure 3B], with the fewest alterations in Citrate lEVs. Remarkably, the EV-derived protein yield from Norgen tubes was consistently low around the limit of detection [Figure 3A and B, Supplementary Figure 2A].

As displayed in the normalized heatmap on day 0 for the indicated vesicle type [Figure 3C], the EDTA-, Streck RNA-, and ACD-A-derived samples presented the highest protein yields for lEVs, whereas the highest amount of sEV protein was obtained from the EDTA- and Citrate tubes [Figure 3C].

One major challenge in the identification of proteomic biomarkers from EVs is the copurification of soluble plasma proteins, especially lipoproteins. The observed increase in protein content after storage suggests that additional contaminants, such as lipoproteins or non-physiologic EVs, are generated *ex vivo* after blood collection*.* In accordance with MISEV guidelines^[50-52]^, we therefore proceeded to evaluate the presence and abundance of classical EV markers in EV peak fractions from the six different tube candidates. To mitigate technical variability, we pooled isolated lEVs and sEVs from three healthy individuals per condition [Figure 3D-G, Supplementary Figure 2C-F]. Many markers were not detectable in the 0-day baseline samples but were detectable only after 7 days of storage, suggesting an extra-physiological origin rather than a physiological EV origin [Figure 3D-G]. Markers derived from peripheral blood, which are likely generated *ex vivo,* were particularly pronounced in sEVs after 7 days, with strong increases observed for CD235, a red blood cell marker; CD41, a platelet marker; and CD45, a white blood cell marker [Figure 3E and G]. The high signals of these markers in lEVs from Streck DNA and ACD-A tubes, as well as in sEVs from all the tested tubes, confirmed that the isolated EVs were blood cell-derived and were released *ex vivo* during storage at RT.

On day 7, we observed a clear increase in copurified lipoproteins, particularly in ACD-A lEVs, and in all sEVs, with the smallest effect in Streck RNA sEVs [Figure 3D-G]. This can be explained through cell lysis and the release of lipoproteins *ex vivo*. Alternatively, storage at RT could induce changes within the EV corona that impair the separation of EVs and lipoproteins^[53]^.

By quantifying EV yield over copurified lipoproteins by calculating the ratio of CD9 to ApoA1, ACD-A achieved the best purification score for lEVs and Citrate for sEVs, whereas only Streck RNA tubes performed well in both lEVs and sEVs [Supplementary Figure 2E and F].

In addition to lipoprotein contamination, we assessed the amount of apoptotic bodies and cellular debris after 7 days of storage by quantifying the 2,000 × *g* pellets obtained during initial plasma processing [Figure 1A]. While PAX and Streck DNA tubes increased in this fraction over time, ACD-A, Citrate Norgen, and Streck RNA decreased, with Streck RNA showing the lowest overall levels [Supplementary Figure 2G].

We implemented ultrafiltration as an additional quality control step for baseline samples to increase the protein concentration and enable more reliable detection of common EV markers (CD9, GAPDH, and ALIX) and contamination markers (ApoA1, ApoB, CD235a, CD41, and CD45) in all tubes at baseline [Supplementary Figure 2H and I].

### Temporal and methodological variations in sEV size and integrity

Next, we further investigated the number and size of sEVs via nanoparticle tracking analysis (NTA) following MISEV guidelines^[39]^. We applied this method to verify the results of the protein quantification experiments and to assess the particle size and number over time. Indeed, the particle concentration increased dramatically after 7 days of storage in all the tubes, corresponding to the observed changes in protein yield, ranging from a 7-fold (Norgen) to an 87-fold increase (ACD-A; Figure 4A). The limitations of NTA in quantifying particles in solution, irrespective of integrity, and the lack of information about particle identity prompted us to explore TEM quantification in parallel. We developed an image-based workflow to analyze the size of intact sEVs via transmission electron microscopy (TEM) [Figure 4, Supplementary Figure 3]. This EM-based workflow allowed us to include EV measurements down to 35 nm while enabling visual selection of intact EVs. However, the limitations include poor representation due to the limited number of TEM images and, therefore, a lower overall number of EVs per sample to be analyzed. Assessment of 0-day baseline samples (day 0) revealed that the mean TEM size of sEVs ranged from 51 nm (Streck RNA), 54 nm (EDTA), 55 nm (PAX), and 56 nm (ACD-A) to 72 nm (Streck DNA) and 75 nm (Citrate) [Figure 4B-D, Supplementary Figure 3A and B]. Furthermore, after 7 days of storage at RT, the mean TEM size of the isolated sEVs increased significantly across all the tubes [Figure 4B, Supplementary Figure 3C], which was particularly pronounced in the Streck RNA (light brown) and ACD-A (light green) tubes, with up to 3-fold larger particles [Figure 4B]. The number of detected EVs in the TEM images of Norgen tubes was too low for reliable quantification. Therefore, we excluded Norgen from subsequent analyses.

A direct comparison of NTA- and TEM-measured absolute particle sizes revealed incongruencies with overall smaller sizes in TEM than in NTA [Figure 4D], resulting in a method-dependent EV size difference of 100 nm larger in NTA [Figure 4E], independent of storage time and tube type. The low correlation coefficients between protein and particle size (*R*^2^_TEM_ = 0.7854, *R*^2^_NTA_ = 0.4619; Figure 4E) indicated that, especially when measured by NTA, protein cargo did not closely correlate with sEV size [Figure 4E]. Remarkably, after 7 days of storage, in all but the Streck RNA tubes, and most significantly in the PAX and Streck DNA tubes, sEV isolation was accompanied by increased protein yields [Supplementary Figure 3D], as already observed by Western blot [Figure 3D-G]. This finding suggested that the increased amount of protein isolated after seven days of storage reflected a greater degree of coisolated (lipo-)protein rather than isolation of larger EVs carrying increased protein cargo [Supplementary Figure 3E]. In summary, when sEVs were quantified via different approaches, we not only detected an increase in EV number [Figure 4A] but also in size [Figure 4B-D] and the amount of copurified (lipo-)protein [Supplementary Figure 3D] during storage in all preservation tubes. These findings may be explained by *ex vivo* vesicle swelling or additional vesicle release. However, we cannot exclude the possibility that the selective degradation of smaller sEVs may have contributed to the observed increase in size.

### Preservation tube choice impacts the EV proteome

A key to identifying EV-based protein biomarkers is understanding how the quality and representation of isolated EVs affect the proteome. To this end, we performed mass spectrometry on lEVs and sEVs from three healthy individuals, each of which were isolated from different preservation tubes after various storage times. To ensure comparability of EV proteomes after preservation, blood was collected in all tubes at the same time point and either processed immediately or left at RT for 7 days before subsequent isolation.

Assuming that the increase in protein amounts after storage is likely due to non-physiologic EVs generated *ex vivo* and to copurified (lipo-)proteins, we focused the initial analyses of the LC-MS/MS-based proteomics data that were detected on day 0 in any tested tube [Figure 5A]. On day 0, we identified an average of 309-2,205 proteins per tube in lEVs [Supplementary Figure 4A] and 28-1,110 proteins per tube in sEVs [Supplementary Figure 4B], with the lowest counts in Norgen (lEV, *n* = 309; sEV, *n* = 28) and the highest in ACD-A (lEV, *n* = 2,205; sEV, *n* = 1,110). Combining all the tested conditions, we found that 47% of the proteins identified in any tube overlapped across all the tubes in at least one replicate (*n* = 1,830; Figure 5A), with ACD-A tubes containing an exceptionally high number of tube-specific proteins (14%, *n* = 534; Figure 5A). To allow for biological interpretations in the following analyses, we henceforth based our analyses on the iBAQ (intensity-based absolute quantification) values^[31]^, which describe protein quantity adjusted to the size of each identified protein rather than only considering the peptide count [Figure 5B-G, Supplementary Figure 4C-N, Supplementary Figure 5A-C, Supplementary Tables 2-5]. For each iBAQ value, we combined the values of the three individuals as the sum of iBAQ for the indicated condition. Remarkably, after 7 days, we observed an increase in the identified proteins and in the iBAQ values [Figure 5B, Supplementary Figure 4A]. However, this increase in identified proteins and iBAQ values over time was substantially more pronounced in sEVs than in lEVs [Figure 5B, Supplementary Figure 4A]. Overall, the mass spectrometry data were concordant with the protein quantification data obtained with a Qubit instrument [Figure 3A and B, Figure 5B, Supplementary Figure 4A and B].

Owing to the lipoprotein contamination detected by Western blot in the EV samples after 7 days [Figure 3D-G, Supplementary Figure 2C and D], we sought to assign the detected proteins to previously identified EV proteins. We found that of the proteins present in our identified protein list, comprising all proteins detected across the different tubes, 51 were also listed among the top 100 EV proteins from the Vesiclepedia database^[32]^. We plotted the iBAQ values of these 51 EV-annotated proteins with the top 100 EV proteins as an intensity heatmap. Like the identified proteins and protein concentration measurements by Qubit, we observed higher EV protein assignment values in tubes stored for 7 days, with the exception of Norgen, which consistently presented lower values after 7 days [Supplementary Figure 4C and D]. Notably, on day 0, compared with the other samples, the EDTA-derived lEV samples were enriched for EV-associated proteins [Supplementary Figure 4C]. When four selected and well-characterized EV proteins, ALIX, CD9, CD63, and CD81, were examined, we observed the same trend after 7 days. The selected EV proteins were highly represented in lEV samples isolated from EDTA (CD81) and Citrate (CD9 and CD63) on day 0 [Supplementary Figure 4E-L].

In light of the many proteins identified in our analysis not being covered by the top 100 EVs, we next attempted to assess the EV representativeness of our full protein list. We aligned our complete protein list to two more EV databases: proteins of EV origin according to Gene Ontology analysis and those cataloged in the ExoCarta database^[33]^ [Supplementary Figure 4M]. Proteins from our analysis not covered in either of those two databases were further analyzed via the STRING platform^[54]^ and found to localize mainly to the mitochondria [Supplementary Figure 4N]. The reasons for the discrepant detection of mitochondrial proteins in our EV proteomes, in contrast to those in public databases, can be both technical and physiological. One possible explanation is the high sensitivity of our mass spectrometry analysis, as mitochondrial proteins are expressed at low levels in EVs^[55]^. Furthermore, the ExoCarta database is an sEV database, and mitochondrial proteins might be located predominantly in lEVs. This highlights the need for technically well-annotated, comprehensive EV protein databases covering various biological sources and physiological states to improve the baseline framework for future plasma-derived liquid biopsy proteome analyses.

However, assignment to EV databases does not consider whether EVs are generated *ex vivo* or from a physiological source. Therefore, we performed a comprehensive literature search and selected two studies providing comprehensive lists of proteins previously described in blood plasma-derived EVs^[36, 37]^. The systematic review of Vallejo *et al.* provided data from a proteomic meta-analysis^[36]^, whereas the recently published original work by Dhondt *et al.* described proteome data of EVs isolated from different blood preservation tubes^[37]^. First, we investigated the overlap between the previously published protein lists and our proteomic data. For this comparison, we used the complete list of identified proteins in any tested preservation tube (4,395 proteins). Remarkably, there was an overlap of 75% with Vallejo *et al.* (i.e., 1,290 of 1,717 EV-specific proteins identified by Vallejo *et al.* are present in our analysis) and an overlap of 16% with Dhondt *et al.* (i.e., 114 of 710 EV-specific proteins) [Supplementary Figure 5A]. Using the most stringent plasma-derived EV protein list, which was defined as overlap between our study and at least one other study (*n* = 1,346 proteins, Supplementary Figure 5A), we performed t-distributed stochastic neighbor embedding (t-SNE) from this consensus protein list [Supplementary Figure 5B]. The distribution of all samples, independent of individual participants, suggested comparable physiological conditions [Supplementary Figure 5B]. Instead, the main factor of clustering was the preservation tube with Streck RNA for 7 days, and ACD-A and Citrate at both time points formed a separate cluster that included both lEVs and sEVs [Supplementary Figure 5C].

Nevertheless, given the minimal overlap between the protein lists of Vallejo *et al.* and Dhondt *et al.*, we aimed to investigate the complete list of proteins identified in our study for unbiased exploration. To this end, we clustered the iBAQ values of the detected proteins for all tested conditions (tube, storage time, and EV size) and participants via t-SNE [Figure 5C and D]. The samples clustered irrespective of the individual participant [Figure 5C] and Streck RNA (7 days), and Citrate and ACD-A formed separate clusters at both time points [Figure 5D]. Furthermore, this analysis enabled the distinction between lEV and sEV protein cargo across tubes [Figure 5D].

The t-SNE is limited to the observation of distinct clusters without allowing performance assessment. Therefore, we investigated the tissue and functional origins of the respective EVs to uncover potential contamination and preanalytical biases introduced after sample collection in each of the tested tubes [Figure 5E-G, Supplementary Figure 5D-F]. To evaluate the impact of anticoagulants, we directly compared the representations of the tissue proteomes [Figure 5E and F, Supplementary Table 3]. The most prominent and consistent pattern in all tubes was the dramatic, time-dependent increase in peripheral blood-derived proteins, with varying ratios assigned to the bone marrow (cell proliferation, innate immune response; Figure 5E and F; blue) and liver (plasma proteins; Figure 5E and F; green). Owing to the low number of proteins isolated from Norgen tubes, these samples were exempt from this observation. On a smaller scale, sEVs tended to present more spleen- and liver-derived proteins, while lEVs appeared to exhibit more brain-neuronal signaling signatures [Supplementary Figure 5D and E]. The tissue origin of EV proteins was more dependent on EV size, preservation tube, and storage time than on individual participant differences [Supplementary Figure 5D-F].

Direct comparison of the logarithmic-fold changes in contaminating *ex vivo*-generated peripheral blood-derived signatures revealed that the acquisition of non-physiological EV-derived proteins with extended storage times was more pronounced in sEVs than in lEVs, confirming previous findings [Figure 5G]. Streck RNA (sEVs) and Citrate (lEVs) showed the lowest alterations after storage [Figure 5G]. Overall, following the ranking for lEVs and sEVs, Streck RNA, ACD-A, and Citrate, which clustered together in the t-SNE, were characterized by the lowest increase in contamination, followed by PAX and Streck DNA [Figure 5G]. After 7 days of storage, both lEVs and sEVs gained proteins that accounted for bone marrow cell proliferation [Figure 5E-G]. Compared with lEVs, sEVs presented a greater increase in the bone marrow immune response signature [Figure 5G].

To further investigate whether cell death contributes to the observed increase, we analyzed a curated list of apoptosis-associated proteins [Supplementary Figure 5G, Supplementary Table 6]. While apoptotic marker proteins were detectable in both EV types, their increase with storage was considerably weaker than the general increase in iBAQ, as shown in Figure 5B. Overall, lEVs contained higher levels of apoptotic markers than sEVs did, and both EV types presented the greatest increase in apoptotic proteins in ACD-A tubes after 7 days of storage [Supplementary Figure 5G].

We aimed to categorize the proteins detected under each condition based on MISEV protein characterization^[52]^ [Supplementary Figure 5H, Supplementary Tables 7 and 8]. In terms of quantity, we observed the same patterns as previously described [Figure 5]. Interestingly, functional annotation revealed that lEVs presented higher iBAQ values for integrins than sEVs did. Additionally, when the membrane topology of lEVs was analyzed, the presence of single-pass transmembrane proteins was greater than that of sEVs. When we directly compared the storage-related increase in membrane-associated and cytosolic EV proteins, we found that the number of cytosolic proteins in sEVs was greater than the number of membrane proteins in lEVs. This trend was consistent across most tube types, except for Streck DNA, where membrane-associated proteins in lEVs showed a relatively greater increase [Supplementary Figure 5I, Supplementary Table 10].

### EV isolation protocol for serial isolation of cfDNA

**Figure 6:**
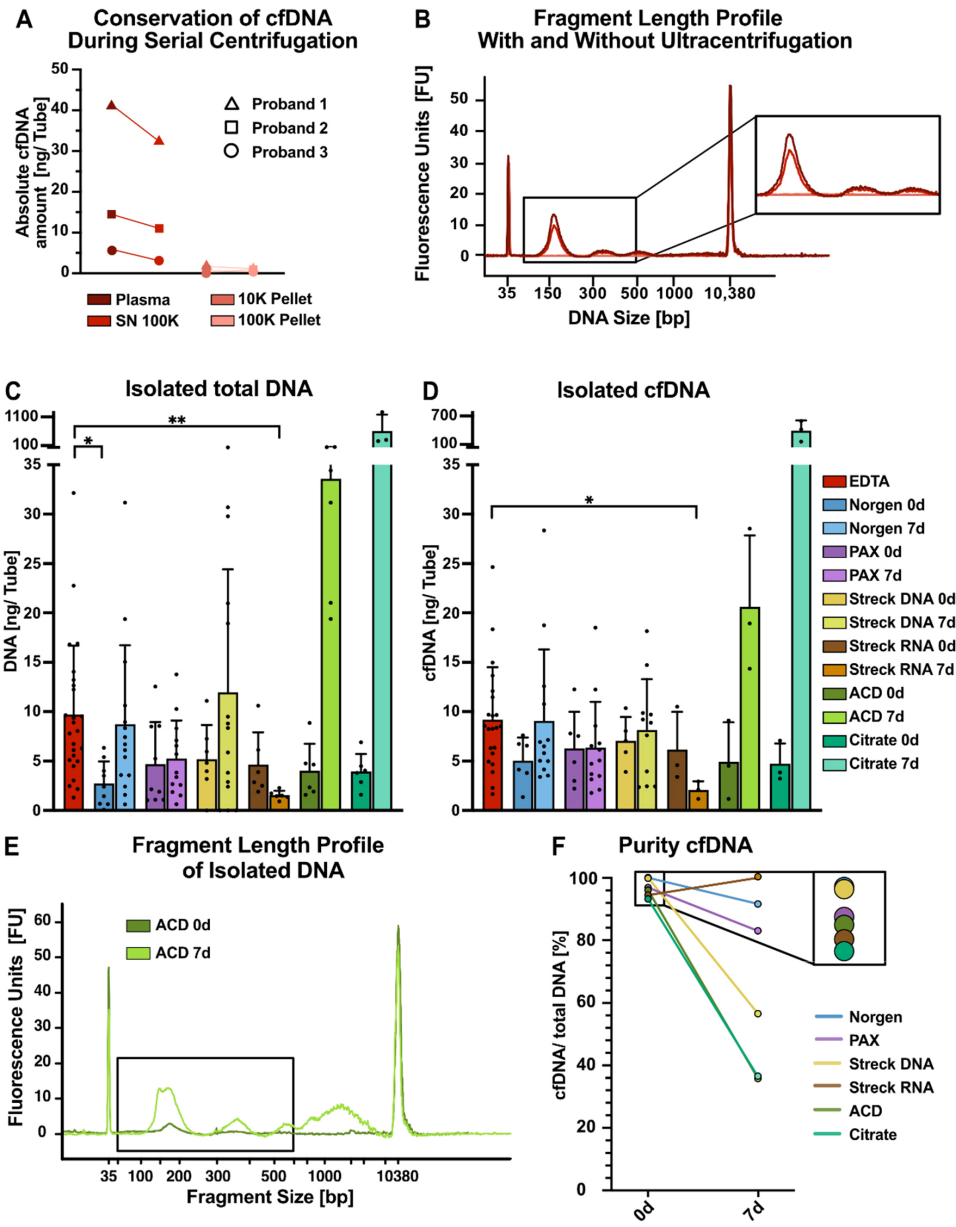
Serial isolation of cfDNA and EVs from tube candidates. (A) Absolute cfDNA amounts measured with Qubit DNA quantification for three individuals; (B) Bioanalyzer (BA) profiles of representative individuals in the indicated plasma fractions from EDTA tubes, e.g., in plasma before ultracentrifugation (dark red), in plasma supernatant after 100 K ultracentrifugation (red), in the 10 K pellet (pink), and in the 100 K pellet (light pink); (C) Absolute amounts of total DNA and (D) cfDNA per tube recovered from 100 K supernatants from the indicated tubes. DNA was isolated via NucleoSnap, and cfDNA was quantified via BA profiles. The size of cfDNA wrapped around one nucleosome can range from approximately 146 to 176 bp and is represented by the first peak at approximately 160 bp; the second and third peaks are represented by multiples thereof; (E) Exemplary BA profiles for DNA isolated from ACD tubes after immediate processing (dark green) and after 7 days (light green), highlighting the increase in genomic DNA contamination over time. The black box indicates the expected size of cfDNA; (F) cfDNA purity of DNA isolations defined as the ratio of cfDNA (first nucleosomal peak) to total DNA (50-7,000 bp) measured by BA. Statistical significance in (C) and (D) was determined for all tube candidates via the Kruskal-Wallis test, followed by Dunn’s multiple comparisons test. ^*^P < 0.05, ^**^P < 0.01. Only statistically significant differences compared with EDTA are shown in the graphs; nonsignificant results are not marked. cfDNA: Cell-free DNA; EVs: extracellular vesicles; lEVs: large EVs; sEVs: small EVs.

One of the advantages of EV isolation via differential ultracentrifugation is the possibility of coisolating cfDNA as a complementary genetic biomarker. To assess the effects of ultracentrifugation on cfDNA yield and quality, we investigated how the initial ultracentrifugation steps impacted the quantity of isolated cfDNA [Figure 6A and B]. After the ultracentrifugation steps (10 and 100 K) required for lEV and sEV separation, we observed a marginal decrease in the amount of cfDNA isolated from the EDTA plasma of three healthy individuals [Figure 6A]. In contrast to previous reports^[56]^, 10 K and 100 K pellets recovered only dismal amounts of DNA from healthy individuals [Figure 6A]. In contrast to the findings of previous miRNA studies, fragment length analyses of isolated DNA with and without ultracentrifugation revealed no apparent changes in the size profiles and confirmed minor reductions in the amount of cfDNA [Figure 6B]^[57]^. An analysis of total DNA and cfDNA amounts isolated from 100 K supernatants from the different tubes revealed that EDTA resulted in the highest yield, but no major differences between the other tubes were observed on day 0 [Figure 6C and D]. While storage at RT for 7 days did not significantly change the amount of cfDNA isolated from PAX tubes, the amount of cfDNA increased slightly in Norgen and Streck DNA tubes over time [Figure 6C and D]. In contrast, the amount of cfDNA in Streck RNA tubes decreased after seven days of preservation. The most dramatic increase in the amount of cfDNA was observed in the plasma from the ACD-A and Citrate tubes after 7 days [Figure 6C and D].

Highlighting an exemplary fragment length profile of ACD-A-derived DNA on day 0 and after 7 days of storage revealed an increase in fragments not only at the size expected for cfDNA (approximately 160 bp and 320 bp) but also longer than 1,000 bp, indicating an increase in genomic DNA contamination, most likely due to a leukocyte burst [Figure 6E], as described previously^[58]^. Finally, we employed the proportion of cfDNA relative to total DNA as a surrogate for cfDNA purity [Figure 6F]. When processed immediately, the highest cfDNA purity was achieved from Norgen tubes, followed closely by Streck DNA tubes [Figure 6F]. Overall, only minor differences in purity were observed for the 0-day baseline samples. However, when blood was stored at RT for 7 days, dramatic differences in cfDNA purity were observed depending on the preservation tube. The purity of cfDNA in Streck RNA tubes was greater than that in immediate processing tubes, whereas the purities in Norgen and PAX tubes were moderately lower and dramatically lower for ACD-A, Citrate, and, to some extent, Streck DNA [Figure 6F].

## DISCUSSION

In this work, we compared the performance of seven commercially available blood preservation tubes for comprehensive liquid biopsy studies, including proteome analyses of lEVs and sEVs coisolated along with cfDNA. Although our study was limited by sample size and the specific study population (ten healthy individuals, eight women and two men aged between 23 and 48 years), the results were consistent enough to draw conclusions regarding the performance of different tubes for EV preservation.

Hemolysis levels are a key parameter of quality control, with the highest degrees of hemolysis observed in Norgen and PAX tubes and, to some extent, in Streck DNA. Hemolysis was much exacerbated with extended storage time at RT. However, one must keep in mind that our hemolysis assessment was solely based on photometric properties at 414 nm, which is a valid and sensitive method of detecting hemolysis via free hemoglobin but might lack some specificity.

Another limitation of our study is the exclusive use of a single EV purification method, potentially introducing bias related to ultracentrifugation or size exclusion chromatography using IZON columns. However, our findings align with recent studies that have evaluated preservation tube performance via alternative EV isolation and analysis approaches^[37]^. However, the proper choice of sampling tubes and timely downstream processing are crucial for ensuring the integrity of the results.

By assessing the quality and quantity of the EV isolates, we observed an increase in total protein levels after 7 days of storage at RT across all the tested tubes. This increase was attributed primarily to *ex vivo*-generated EVs, which we found in our EV isolates. Furthermore, the protein quantification results clearly indicated that Norgen tubes are unsuitable for EV analysis, as they yielded notably low protein amounts even on day 0.

We performed systematic size analysis of the isolated vesicles via two independent methods, namely NTA and TEM. First, while the trends were comparable, our data suggest an overestimation of vesicle size by NTA. Possible explanations are the overestimation of EVs by detection of larger protein complexes, the higher detection limit of NTA excluding very small vesicles less than 100 nm, and the fact that NTA measures the hydrodynamic diameter rather than the geometric size of the vesicles^[59]^. In contrast, TEM preparation can influence the integrity of EVs and image quality^[60]^. However, there are no data supporting vesicle shrinkage upon negative transmission electron microscopy (TEM) or size modulation based on imaging when instrument calibration is frequently performed. However, we observed a significant increase in particle size with increasing storage time with both methods. This increase could be explained by an unspecific fusion or swelling of EVs that has been described to occur over time, even under conditions of freezing to -20 or -80 °C^[61]^. Second, EVs released by blood cells *ex vivo* are described to be larger than physiological EVs, thereby shifting the mean size to higher values^[62]^.

Compared with PAX and Streck DNA tubes, MS-based proteomic data suggested superior performance of Streck RNA, ACD-A, and Citrate tubes at both time points and for both EV subtypes. This method also revealed a high overlap of detected proteins in our samples with EV databases, validating our EV isolation workflow. On this basis, we discovered many novel EV-associated proteins, many of which have not been previously described, highlighting the comprehensive nature of our data set. In the future, our data, especially the differential hits observed between the 0-day baseline and 7-day samples, might therefore become the basis for a database of *ex vivo*-generated EV-derived proteins. This database could function as a reference resource for proteomic EV biomarker studies, enabling the preparation of more clinical EV diagnostic tools, such as the ExoDx prostate test^[63]^. This EV test showed improved risk stratification for prostate cancer compared with the standard of care, reducing unnecessary biopsies and detecting more high-grade cancers.

Additionally, we demonstrated that cfDNA can be coisolated along with EVs and that the ultracentrifugation steps required for EV purification do not compromise the cfDNA yield or integrity. Despite their strong performance in preserving EVs with minimal hemolysis and low contamination, the ACD-A and Citrate tubes presented substantial genomic DNA contamination. As a result, they are less suitable for cfDNA or multiomics studies but remain viable options for proteomic EV analyses. Notably, Citrate tubes offer practical advantages over ACD-A tubes, including greater cost-effectiveness and broader availability in clinical practice.

The key strength of our study lies in the comprehensive assessment of tube performance for the isolation of lEVs, sEVs, and cfDNA, thereby providing insights into their suitability for multiomics applications in clinical settings.

Taken together, for multiomics liquid biopsy diagnostics, Streck RNA tubes achieved the highest quality score, despite increased amounts of *ex vivo*-generated EV-derived proteins after 7 days. Compared with the other tube candidates, these candidates exhibited a low degree of hemolysis and good EV quality, with only minor contamination with lipoproteins or red blood cell-/platelet-derived material, while at the same time conserving high-purity cfDNA. In conclusion, Streck RNA tubes are the preferred option for the simultaneous isolation of cfDNA and EV-derived proteins.

## CONCLUSION

In this study, concurrent cfDNA- and EV-based liquid biopsies from seven different types of blood preservation tubes were compared. As a consequence, upcoming clinical studies can make well-informed sampling choices depending on clinical questions, logistics, and preferred biomarkers, with Streck RNA tubes providing the most universal biomarker conservation.

## DECLARATIONS

### Availability of data and materials

All the raw mass spectrometry data were uploaded to the PRIDE server under the accession code PXD062837.

### Copyright

© Author(s) 2025.
