# Supplementary material for: Preanalytical framework for routine clinical use of liquid biopsies: combining EVs and cfDNA
Source: Extracell Vesicles Circ Nucl Acids. 2025 Oct 9;6(4):626–50. doi: 10.20517/evcna.2025.44 (PMC12809394; doi:10.20517/evcna.2025.44)
Supplement: Supplementary file 1 [file evcna-6-4-626-SupplementaryMaterials.zip › evcna6044-SupplementaryMaterials/Supplementary Table 1, Supplementary Figures 1-5.pdf]

Supplementary Materials

Preanalytical framework for routine clinical use of liquid biopsies: combining EVs and cfDNA

Nike K. Simon[1,2,3], Stefanie Volz[1,2,4], Jussara Rios de los Rios Reséndiz[5,6], Tatjana Wedig[1,2,4], Sophia H. Montigel[1,2,3], Nathalie Schwarz[1,2], Karsten Richter[7], Dominic Helm[8], Michelle Neßling[7], Lin Zielske[5], Julia Berker[5,6], Sophia Russeck[5,6], Monika Mauermann[1,2], Wolf-Karsten Hofmann[6], Stefan M. Pfister[1,2,3,4], Kristian W. Pajtler[1,2,3,4], Kendra K. Maaß[1,2,3,4], Katharina Clemm von Hohenberg[5,6]

[1]Division of Pediatric Neurooncology, German Cancer Consortium (DKTK), German Cancer Research Center (DKFZ) Heidelberg, Heidelberg 69120, Germany.

[2]Hopp Children's Cancer Center Heidelberg (KiTZ) and NCT Heidelberg, Heidelberg 69120, Germany.

[3]Medical Faculty, Heidelberg University, Mannheim 68167, Germany.

[4]Department of Pediatric Oncology, Hematology and Immunology, Heidelberg University Hospital, Heidelberg 69120, Germany.

[5]Junior Clinical Cooperation Unit Translational Lymphoma Research, German Cancer Research Center (DKFZ) Heidelberg, Mannheim 68169, Germany.

[6]Department of Hematology and Oncology, Medical Faculty Mannheim, Heidelberg University, Mannheim 68167, Germany.

[7]Core Facility Electron Microscopy, German Cancer Research Center (DKFZ) Heidelberg, Heidelberg 69120, Germany.

[8]Proteomics Core Facility, German Cancer Research Center (DKFZ) Heidelberg, Heidelberg 69120, Germany.

Correspondence to: Dr. Kendra K. Maaß, Junior Group Prevention and Liquid Biopsies, Hopp-Kindertumorzentrum Heidelberg (KiTZ), Heidelberg 69120, Germany. E-mail: k.maass@kitz-heidelberg.de; Dr. Katharina Clemm von Hohenberg, Junior Clinical Cooperation Unit Translational Lymphoma Research, German Cancer Research Center (DKFZ) Heidelberg, Pettenkoferstr. 22, Mannheim 68169, Germany. E-mail: k.clemm@dkfz-heidelberg.de

Supplementary Table 1. Characteristics of blood preservation tubes and plasma isolation parameters.

Tube	EDTA	Norgen	PAX	Streck DNA	Streck RNA	ACD	Citrate
Function	Hematological studies	DNA, RNA	DNA, RNA	DNA	RNA, EV	Blood grouping test and cell preservation	Coagulation studies
Volume	9	8,4	10	10	10	9	9
Storage Time	Max 1h	30 days at RT	14 days at RT	14 days at RT	7 days	21 days	4h
Additive	Ca Ions complexation	Osmotic cell stabilization	Biological apoptosis prevention	Chemical crosslinking	Chemical crosslinking	Acid citrate dextrose	3,2 % sodium citrate
Centrifugation	10 min 1900 x g 4 °C	20 min 500 x g RT	15 min 1900 x g RT	15 min 1600 x g RT	10 min 2500 x g	10 min 2500 x g	10 min 2000 x g

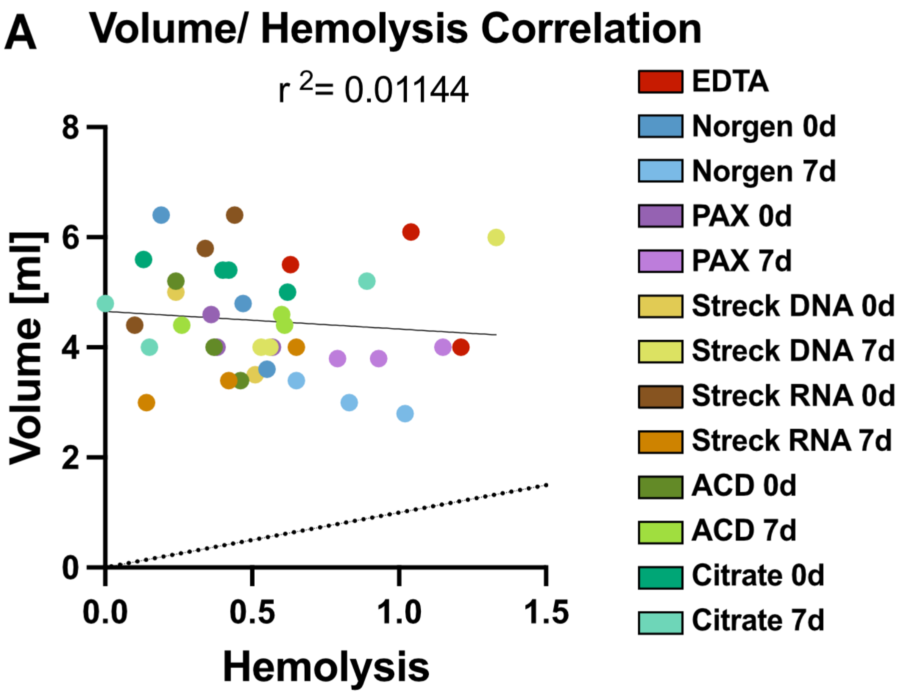

Supplementary Figure 1. Correlation of volume to hemolysis in different tube candidates **(A)** Correlation of plasma volume recovered from tube candidates after centrifugation and hemolysis levels determined by relative levels of free hemoglobin measured as blood plasma absorbance at 414 nm. The linear regression line is shown with 95 % confidence intervals, R^2 as depicted, P= 0.5168.

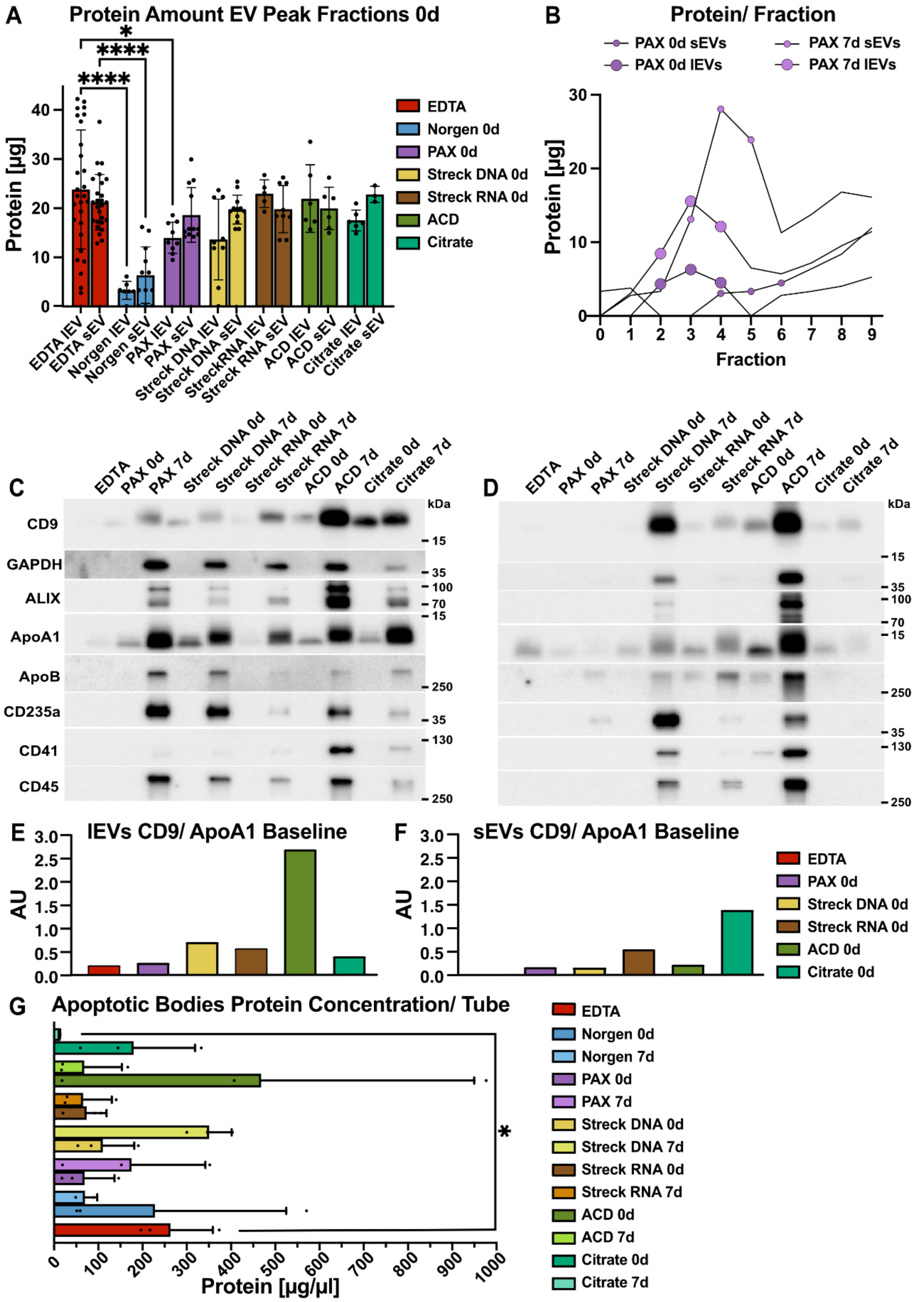
A
Protein Amount EV Peak Fractions 0d
Protein [µg]
EDTA
Norgen 0d
PAX 0d
Streck DNA 0d
Streck RNA 0d
ACD
Citrate
EDTA lEV
EDTA sEV
Norgen lEV
Norgen sEV
PAX lEV
PAX sEV
Streck DNA lEV
Streck DNA sEV
StreckRNA lEV
Streck RNA sEV
ACD lEV
ACD sEV
Citrate lEV
Citrate sEV
B
Protein/ Fraction
PAX 0d sEVs
PAX 0d lEVs
PAX 7d sEVs
PAX 7d lEVs
Protein [µg]
Fraction
C
EDTA
PAX 0d
PAX 7d
Streck DNA 0d
Streck DNA 7d
Streck RNA 0d
Streck RNA 7d
ACD 0d
ACD 7d
Citrate 0d
Citrate 7d
kDa
CD9
GAPDH
ALIX
ApoA1
ApoB
CD235a
CD41
CD45
D
E
lEVs CD9/ ApoA1 Baseline
AU
F
sEVs CD9/ ApoA1 Baseline
EDTA
PAX 0d
Streck DNA 0d
Streck RNA 0d
ACD 0d
Citrate 0d
G
Apoptotic Bodies Protein Concentration/ Tube
Protein [µg/µl]
EDTA
Norgen 0d
Norgen 7d
PAX 0d
PAX 7d
Streck DNA 0d
Streck DNA 7d
Streck RNA 0d
Streck RNA 7d
ACD 0d
ACD 7d
Citrate 0d
Citrate 7d

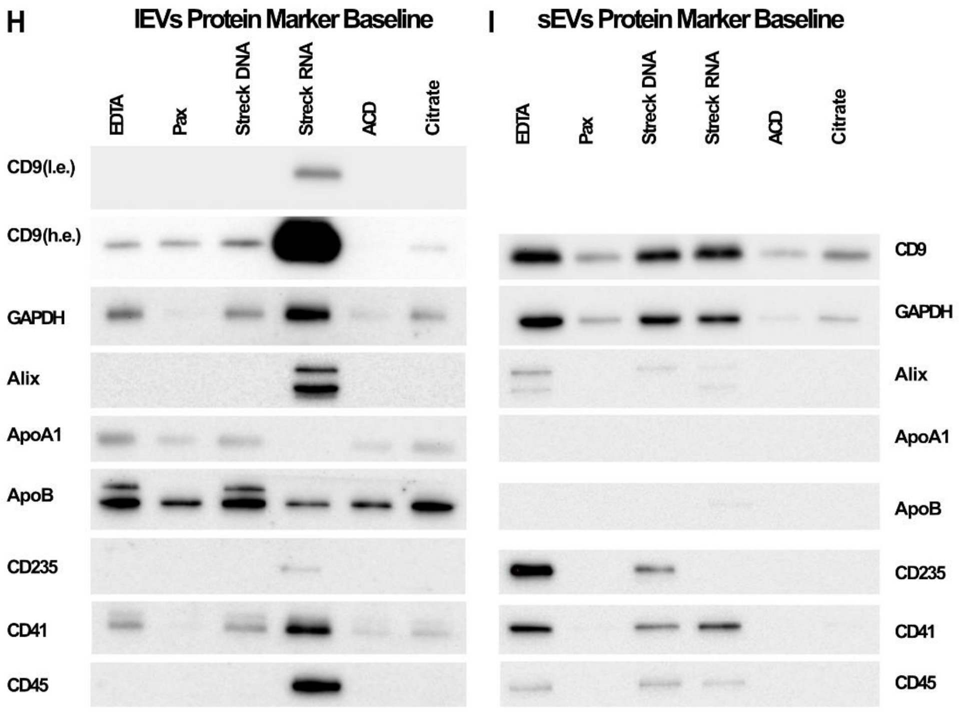

Supplementary Figure 2. Quantitative analysis of lEVs and sEVs **(A)** lEV and sEV protein amounts in three peak fractions per tube after immediate processing. Underlying data is identical to Figure 3A, B. **(B)** Exemplary protein amount profile of SEC fractions lEV and sEV collected from PAX tubes after immediate processing and after 7d highlighting the selection of the three peak fractions for lEVs 0 days (Fr. 2-4), lEVs 7 days (Fr. 2-4), sEVs 0 days (Fr. 4-6), and sEVs 7 days (Fr. 3-5). Soluble proteins are eluted in later fractions (lEVs Fr.6-9, sEVs Fr. 7-9). quantified from Western Blot data**. (C, D)** Western Blot analyses of pooled lEVs' and sEVs' peak fractions from three healthy individuals for classical EV marker, lipoprotein contamination and blood-derived EV marker with protein ladder sizes indicated. **(G)** Protein concentration of apoptotic bodies (2,000xg pellet) at baseline and after 7 days measured with BCA. **(H, I)** Western Blot analyses of lEVs' and sEVs' peak fractions after concentration by ultrafiltration. **(E,F)** Relative abundance of the EV marker CD9 and of ApoA1 as a marker for lipoprotein contamination Statistical significance in **(A)** and **(G)** was determined using Kruskal–Wallis test, followed by Dunn's multiple comparisons test. * $P < 0.05$, **** $P < 0.0001$. Only statistically significant differences compared to EDTA are shown in the graphs; non-significant results are not marked. Norgen was excluded from the Suppl. Figure SS C, D because it fell below the threshold due to the limited sample amount. One biological replicate of Norgen 7d was excluded in Suppl. Figure S2G due to insufficient material after isolation.

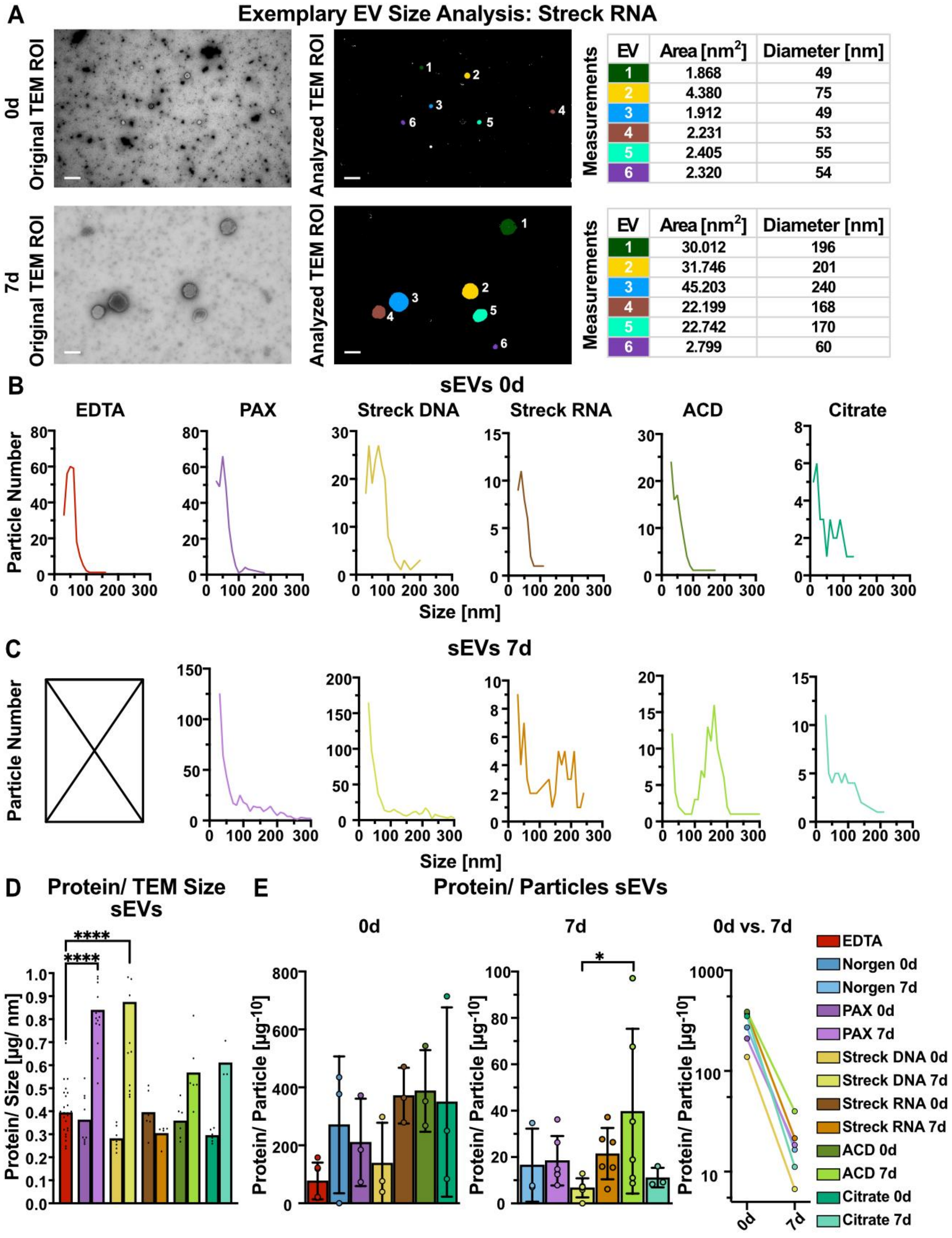

Supplementary Figure 3. Methodological comparison of size and integrity of sEVs (A) TEM image analysis of EV size of representative Streck RNA samples. Transmission electron microscopy (TEM) images were taken after immediate processing (top) and after 7d of preservation (bottom). TEM images (left) were analyzed using ilastik version 1.4.0 software to create probability maps. ImageJ was used for quantification and measurements, generating a table with depicted parameters for detected EVs (right) and

number traceable in the image (middle) (Scale bar = 200 nm). **(B-C)** Particle size distribution of sEVs after immediate processing **(B)** and after 7d **(C)** is plotted as sEV diameter measured from TEM negative stainings. **(D)** Ratio of protein per TEM sizes. **(E)** Ratio of protein per particle for each tube at baseline (left), after 7 days (middle) and time-dependent changes (right). Statistical significance for **(D)** was determined using Kruskal–Wallis test, followed by Dunn's multiple comparisons test * $P < 0.05$, **** $P < 0.0001$. Only statistically significant differences compared to EDTA are shown in the graphs; non-significant results are not marked.

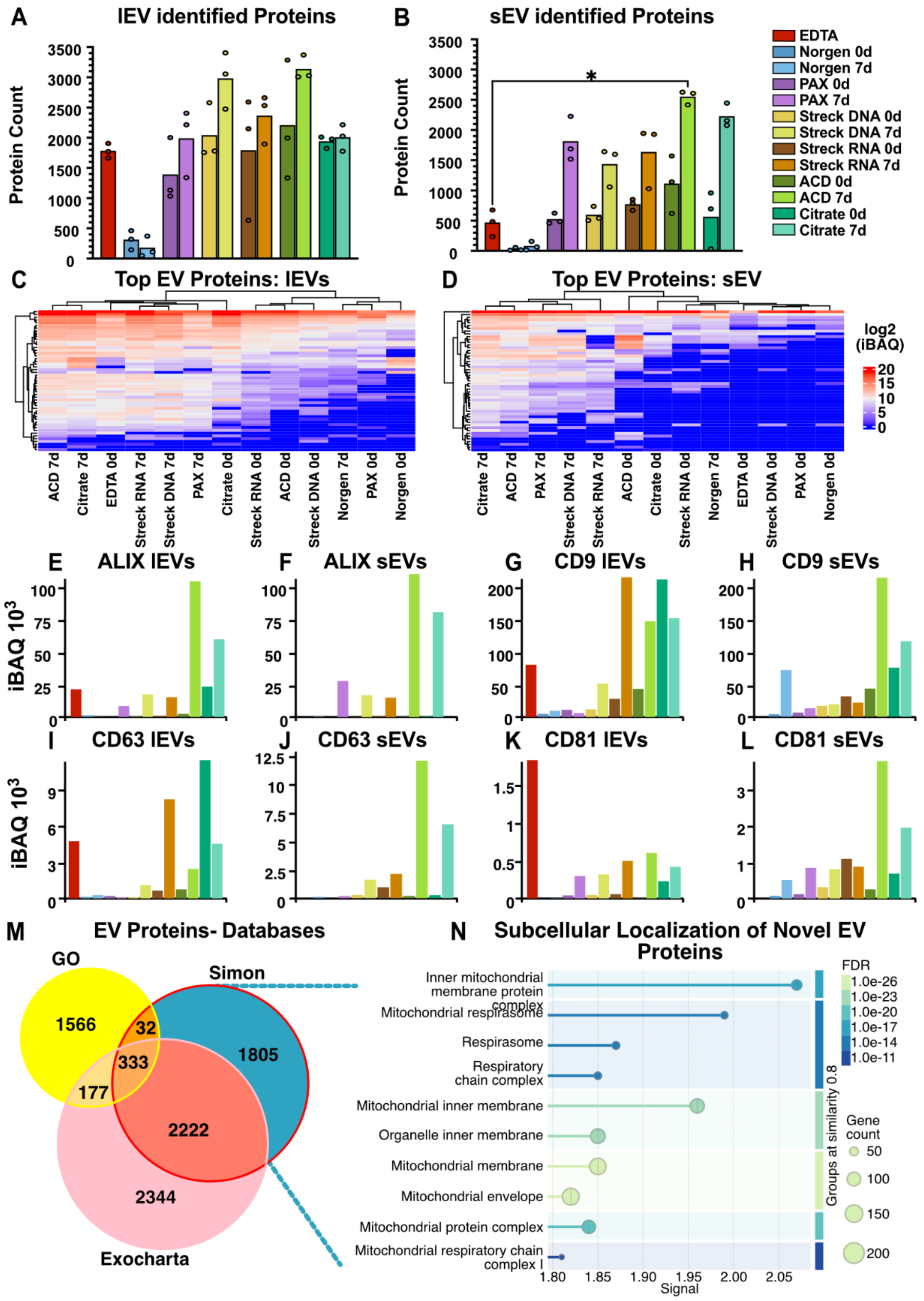

Supplementary Figure 4. Comprehensive proteome characterization of lEVs and sEVs. **(A-B)** Absolute numbers of identified proteins in lEV **(A)** and sEV **(B)** peak fractions. **(C)** lEVs and **(D)** sEVs heatmaps displaying the summed iBAQ values for indicated tube types and preservation times of 51 proteins abundant in our samples that overlap with the top 100 EV proteins according to the Vesiclepedia database(1). **(E-L)** Summed iBAQ values of all lEV and sEV samples of indicated tube types at baseline and after 7d for ALIX **(E, F)**, CD9 **(G, H)**, CD63 **(I, J)**, CD81 **(K, L)**. **(M)** Venn diagram illustrating the number of identified proteins by mass spectrometry and their overlap with GO Term and Exocharta(2) EV

protein lists. **(N)** STRING(1, 3) analysis of the subcellular localization of proteins not overlapping with neither GO term nor Exocharta EV protein list. Statistical significance for **(A)** and **(B)** was determined using Kruskal–Wallis test, followed by Dunn's multiple comparisons test * $P < 0.05$

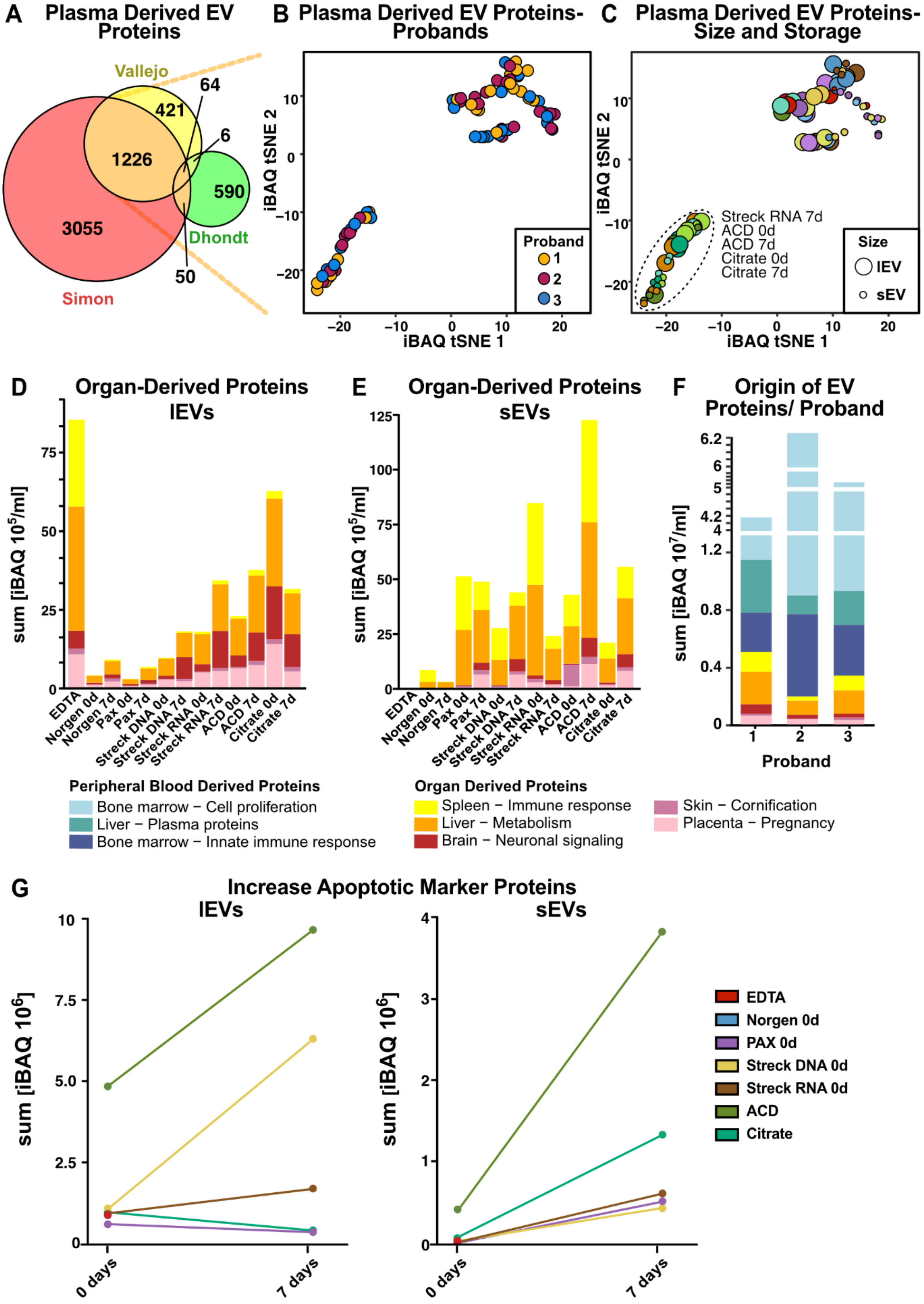

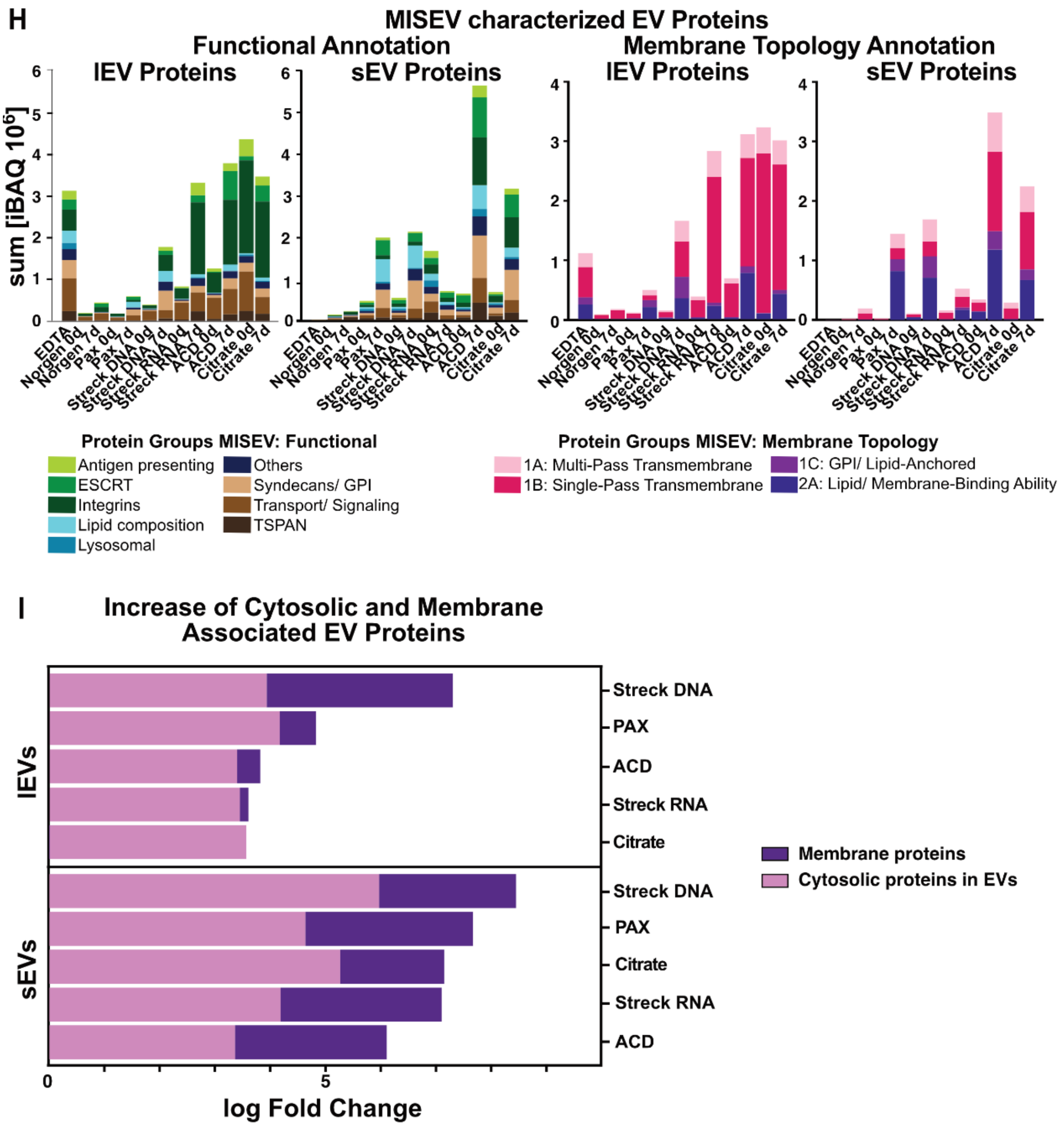

Supplementary Figure 5. Deciphering EV protein origin by mass spectrometry **(A)** Venn diagram illustrating the number of proteins by mass spectrometry and their size-adjusted overlap with Vallejo *et al.*(4) and Dhondt *et al.*(5). **(B, C)** t-SNE clustering of three different probands **(B)** and different tube types, sizes and storage times **(C)** depicting plasma derived lEV and sEV proteins defined by the overlap of proteins in all EV protein lists (Vallejo, Dhondt and Simon, indicated in **(A)**). **(D, E)** Organ-derived origin estimation of proteins identified in **(B)** lEVs and **(C)** sEVs. **(F)** Tissue origin estimation of identified proteins in three different probands in all tubes. **(G)** Increase of apoptotic marker proteins iBAQ values over time. **(H)** Functional annotation of proteins according to MISEV(6) protein characterization for lEVs and sEVs (left) and membrane topology annotation according to MISEV(6) protein characterization for lEVs and sEVs (right). **(I)** iBAQ log fold change of transmembrane (or GPI−anchored) proteins associated with plasma membrane and/or endosomes proteins (dark purple) and cytosolic EV proteins (light purple) over storage for 7d vs. 0d. Norgen was excluded from the Fig. S5H, because it fell below the threshold due to the limited protein amount isolated.

REFERENCES

1. Chitti SV, Gummadi S, Kang T, Shahi S, Marzan AL, Nedeva C, et al. Vesiclepedia 2024: an

extracellular vesicles and extracellular particles repository. Nucleic Acids Res. 2024;52(D1):D1694-d8. doi: 10.1093/nar/gkad1007. PubMed PMID: 37953359; PubMed Central PMCID: PMC10767981.

2. Mathivanan S, Fahner CJ, Reid GE, Simpson RJ. ExoCarta 2012: database of exosomal proteins, RNA and lipids. Nucleic Acids Res. 2012;40(Database issue):D1241-4. Epub 20111011. doi: 10.1093/nar/gkr828. PubMed PMID: 21989406; PubMed Central PMCID: PMC3245025.
3. Szklarczyk D, Kirsch R, Koutrouli M, Nastou K, Mehryary F, Hachilif R, et al. The STRING database in 2023: protein-protein association networks and functional enrichment analyses for any sequenced genome of interest. Nucleic Acids Res. 2023;51(D1):D638-D46. doi: 10.1093/nar/gkac1000. PubMed PMID: 36370105; PubMed Central PMCID: PMC9825434.
4. Vallejo MC, Sarkar S, Elliott EC, Henry HR, Powell SM, Diaz Ludovico I, et al. A proteomic meta-analysis refinement of plasma extracellular vesicles. Sci Data. 2023;10(1):837. Epub 20231128. doi: 10.1038/s41597-023-02748-1. PubMed PMID: 38017024; PubMed Central PMCID: PMC10684639.
5. Dhondt B, Pinheiro C, Geeurickx E, Tulkens J, Vergauwen G, Van Der Pol E, et al. Benchmarking blood collection tubes and processing intervals for extracellular vesicle performance metrics. J Extracell Vesicles. 2023;12(5):e12315. doi: 10.1002/jev2.12315. PubMed PMID: 37202906; PubMed Central PMCID: PMC10196222.
6. Welsh JA, Goberdhan DCI, O'Driscoll L, Buzas EI, Blenkiron C, Bussolati B, et al. Minimal information for studies of extracellular vesicles (MISEV2023): From basic to advanced approaches. J Extracell Vesicles. 2024;13(2):e12404. doi: 10.1002/jev2.12404. PubMed PMID: 38326288; PubMed Central PMCID: PMC10850029.
